# Huayu Wan induces ferroptosis and enhances anti-PD-1 efficacy in cisplatin-resistant esophageal squamous cell carcinoma

**DOI:** 10.3389/fimmu.2026.1967436

**Published:** 2026-10-27

**Authors:** Ting Zhu, Bin Wang, Haiyong Wang, Jianyi Ding, Yangjun Xu, Jianfeng Xu, Lingjun Dong, Xiang Wang, Guangmao Yu, Zhupeng Li

**Affiliations:** 1 Department of Thoracic Surgery, Shaoxing People’s Hospital, School of Medicine, Shaoxing University, Shaoxing, China; 2 Department of Cardiac and Great Vascular Surgery, Shaoxing People’s Hospital, School of Medicine, Shaoxing University, Shaoxing, China

**Keywords:** anti-PD-1 therapy, cisplatin resistance, esophageal squamous cell carcinoma, ferritinophagy, ferroptosis, Huayu Wan

## Abstract

Cisplatin resistance remains a major therapeutic challenge in esophageal squamous cell carcinoma (ESCC). Here, we investigated the antitumor effects and underlying mechanisms of Huayu Wan (HYW) in cisplatin-resistant ESCC. HYW markedly inhibited the growth of cisplatin-resistant human KYSE150 and murine mEC25 cells and induced a ferroptotic phenotype characterized by reactive oxygen species accumulation, increased intracellular iron, glutathione depletion, lipid peroxidation, and reduced GPX4 expression. Ferrostatin-1 partially reversed HYW-induced cytotoxicity. HYW also promoted immunogenic cell death-associated changes, including extracellular ATP and HMGB1 release and cell-surface calreticulin exposure. Mechanistically, HYW increased p53 expression and impaired the SLC7A11/GCLC-dependent cystine–glutathione antioxidant system, whereas GCLC overexpression and P53 inhibition partially restored redox homeostasis and cell survival. In parallel, HYW suppressed AKT–mTOR signaling and promoted NCOA4-associated ferritinophagy, as evidenced by increased NCOA4 and LC3-II and decreased FTH expression. Pharmacological inhibition of autophagy and NCOA4 knockdown attenuated iron accumulation and HYW-induced cytotoxicity. In cisplatin-resistant syngeneic tumors, HYW suppressed tumor growth with generally acceptable systemic tolerability, enhanced ferroptosis- and immunogenic stress-associated changes, reduced PD-L1 expression, and increased CD8^+^ T-cell infiltration. Moreover, HYW combined with anti-PD-1 treatment produced greater antitumor effects than either monotherapy. These findings suggest that HYW promotes ferroptosis through coordinated disruption of glutathione metabolism and NCOA4-associated ferritinophagy while enhancing antitumor immune responses in cisplatin-resistant ESCC.

## Introduction

Esophageal squamous cell carcinoma (ESCC) remains a malignancy of profound global aggressiveness, characterized by a high incidence in East Asia and persistently dismal survival rates. While diagnostic and multimodal therapeutic approaches have advanced, outcomes for patients with advanced-stage disease have seen negligible improvement over recent decades (1). Cisplatin-based chemotherapy remains the cornerstone of first-line systemic treatment for advanced ESCC (2). Yet, the efficacy of this regimen is overwhelmingly compromised by the rampant development of intrinsic and acquired resistance, which constitutes the predominant cause of therapeutic failure and disease recurrence (3). Once resistance develops, patients are more likely to experience reduced responses to subsequent treatment and unfavorable survival outcomes. This therapeutic deadlock underscores both the biological complexity of ESCC and the urgent necessity for innovative strategies to overcome cisplatin resistance. Consequently, deciphering the mechanistic drivers of cisplatin resistance and pioneering novel interventions to counteract it are critical and unmet imperatives for altering the therapeutic landscape for ESCC patients.

In recent years, ferroptosis, an iron-dependent and lipid peroxidation-driven form of regulated cell death, has been increasingly recognized as a promising therapeutic avenue in cancer treatment (4, 5). Extensive studies have revealed that ferroptosis profoundly shapes the response of esophageal squamous cell carcinoma to therapy by integrating metabolic reprogramming, redox imbalance, and iron homeostasis. Pharmacologic or genetic activation of ferroptosis can suppress malignant proliferation, amplify oxidative stress, and restore sensitivity to cytotoxic treatments (6–8). Recent studies further demonstrate that both repurposed or synthetic agents and bioactive natural compounds can exploit ferroptotic vulnerabilities in malignancies through distinct molecular mechanisms. For example, the natural compound dioscin induces ferroptosis by suppressing the SLC7A11/GPX4 axis (9), while salinomycin has been shown to promote ferroptotic cell death in multidrug-resistant gastric cancer cells through regulation of ferroptosis-related signaling pathways (10). Moreover, the repurposed antibiotic kitasamycin enhances ferroptosis susceptibility by promoting NCOA4-mediated ferritinophagy and has been reported to improve the efficacy of immune checkpoint blockade (11). Intriguingly, tumor cells that develop resistance to conventional chemotherapeutic agents such as cisplatin often acquire metabolic adaptations that render them more vulnerable to ferroptotic death, implying that ferroptosis may serve as a critical axis to overcome drug resistance (12, 13). Beyond its role as a cell intrinsic death mechanism, ferroptosis exerts broad immunological consequences within the tumor microenvironment. The accumulation of oxidized lipids and the release of damage associated molecular patterns during ferroptosis can enhance antigen presentation, stimulate dendritic cell maturation, and reinvigorate cytotoxic T cell activity, thereby counteracting immune evasion (14, 15). Collectively, these observations underscore ferroptosis as a mechanistically distinct and therapeutically actionable pathway whose deliberate induction may simultaneously reverse chemoresistance and alleviate immunosuppression in esophageal squamous cell carcinoma.

Induction of ferroptosis is a promising avenue to counter chemoresistance, although some current small molecule inducers can present challenges in stability *in vivo*, pharmacokinetics, and selectivity that may temper clinical translation (5). By contrast, traditional multi component formulations offer multitarget regulation with generally good biocompatibility and hold the potential to tune redox balance, lipid metabolism, and immune context in parallel (16). Recent studies have shown that natural products modulate ferroptotic checkpoints, including inhibition of system Xc^−^, suppression of GPX4, disruption of iron homeostasis, and promotion of lipid peroxidation. Previous studies have shown that ferroptosis induction can restore platinum sensitivity (17).

Huayu Wan (HYW), derived from the classical traditional Chinese medicine formulas Taohong Siwu decoction and Didang decoction, is a multi-component formulation and comprises *Amomum villosum* Lour*, Angelica dahurica* (Hoffm.) Benth. & Hook.f. ex Franch. & Sav. [Apiaceae]*, Astragalus mongholicus* Bunge [Fabaceae]*, arthamus tinctorius* L. [Asteraceae]*, Corydalis yanhusuo* (Y.H.Chou & Chun C.Hsu) W.T.Wang ex Z.Y.Su & C.Y.Wu [Papaveraceae]*, Placenta Hominis, Crassostrea gigas* (Thunberg, 1793)*, Curcuma aromatica Salisb.* [Zingiberaceae]*, Glehnia littoralis* (J.G.Cooper) F.Schmidt ex Miq. [Apiaceae]*, Hirudo nipponia* Whitman, 1886*, Paeonia lactiflora* Pall. [Paeoniaceae]*, Erica montis-hominis* E.G.H.Oliv.*, Prunus persica* (L.) Batsch [Rosaceae]*, Silene vulgaris* (Moench) Garcke [Caryophyllaceae], and *Tabanus bovinus* Linnaeus, 1758 (18–20). Botanical names were verified via The World Flora Online (http://www.worldfloraonline.org; accessed March 29, 2026). Traditionally, HYW has been used for the treatment of “Zhengjia” and “Jieju”, which refer to abdominal masses or tumor-like conditions associated with blood stasis, and the principle of “Huoxue Huayu” is considered essential for resolving such pathological masses. Consistent with this traditional indication, HYW has been applied clinically in tumor-related conditions, and a clinical study reported that HYW combined with chemotherapy improved therapeutic outcomes in patients with ovarian cancer (21). Moreover, HYW has been shown to modulate tumor perfusion and enhance the delivery efficiency of chemotherapeutic agents such as doxorubicin, potentially by promoting blood circulation and removing blood stasis (18). Furthermore, HYW was shown to inhibit the growth and metastasis of lung cancer by influencing the platelet pathway (20). In addition, several bioactive components of HYW exhibit anti-tumor activities, including formononetin from Astragalus membranaceus, which inhibits cell cycle progression in esophageal squamous cell carcinoma (ESCC), and extracts of Curcuma zedoaria, which suppress ESCC cell proliferation, invasion, and colony formation (22, 23). Despite these findings, the molecular mechanisms underlying the antitumor effects of HYW in ESCC remain poorly defined, particularly whether HYW can induce ferroptosis and suppress the growth of cisplatin-resistant ESCC.

This study aimed to evaluate the antitumor activity of HYW in cisplatin-resistant ESCC models and to elucidate the underlying mechanisms. We found that HYW suppressed the growth of cisplatin-resistant ESCC cells and tumors. Mechanistically, HYW impaired glutathione metabolism and promoted ferritinophagy-associated iron accumulation, thereby inducing ferroptotic cell death. HYW treatment was also associated with reduced PD-L1 expression, increased CD8^+^ T-cell infiltration, and improved response to PD-1 blockade. These findings identify ferroptosis as a potential therapeutic vulnerability that may be exploited by HYW in cisplatin-resistant ESCC.

## Materials and methods

### Cell lines and reagents

The human esophageal squamous cell carcinoma cell line KYSE150 was obtained from Procell Life Science & Technology Co., Ltd. (Wuhan, China). The mouse esophageal cancer cell line mEC25 was purchased from Otwo Biotech (Guangzhou, China). KYSE150 cells were cultured in RPMI-1640 medium supplemented with 10% fetal bovine serum (FBS) and 2 mM L-glutamine, while mEC25 cells were maintained in DMEM (high glucose) supplemented with 10% FBS. All cells were cultured at 37 °C in a humidified incubator containing 5% CO_2_ and routinely passaged according to the supplier’s recommendations. Primary antibodies against GPX4 (#52455), GAPDH (#9153), p53 (#9282), GCLC (#48005), xCT/SLC7A11 (#12691), NRF2 (#12721), NCOA4 (E8H8Z, #66849), FTH (#3998), LC3B (#2775), AKT (#4691), phospho-AKT (Ser473) (D9E, #4060), mTOR (#2972), phospho-mTOR (Ser2448) (D9C2, #5536), and CD8α (2.43, APC-conjugated, #64786) were purchased from Cell Signaling Technology (Danvers, MA, USA). Recombinant anti-PD-L1 antibody (EPR19759) was obtained from Abcam (Cambridge, UK). InVivoMAb anti-mouse PD-1 (CD279) antibody was purchased from Bio X Cell (Lebanon, NH, USA).

### Cell viability assay

Cell viability was assessed using the Cell Counting Kit-8 (CCK-8; AbMole, Shanghai, China) as previously described (4). Cells were seeded in 96-well plates (3–5 × 10³ cells/well) and treated with the indicated concentrations of HYW for 48 h. Cells were then incubated with 10 μl of CCK-8 solution for 1–2 h at 37 °C. Absorbance at 450 nm was measured using a microplate reader, and cell viability was expressed as a percentage relative to control cells.

### EdU incorporation assay

Cell proliferation was assessed using the Cell-Light™ EdU Apollo488 in Vitro Imaging Kit (RiboBio, Guangzhou, China) according to the manufacturer’s instructions. Briefly, cells were seeded on coverslips in 24-well plates and treated with the indicated concentrations of HYW, with or without the indicated inhibitors, for 48 h. EdU (10 μM) was added to the medium and incubated for 2 h at 37 °C. Cells were then fixed with 4% paraformaldehyde, permeabilized with 0.5% Triton X-100, and stained with the Apollo 488 reaction cocktail to visualize incorporated EdU (green fluorescence). Nuclei were counterstained with DAPI (blue). Fluorescence images were captured with a Leica fluorescence microscope, and the percentage of EdU-positive cells was calculated relative to total DAPI-stained nuclei.

### Colony-formation assay

Cells were seeded into 12-well plates at a density of 1000 cells per well and cultured for 9 days under standard conditions until visible colonies formed. The colonies were then washed twice with PBS, fixed with 4% paraformaldehyde for 15 min, and stained with 0.1% crystal violet for 20 min at room temperature. After washing and air-drying, images were captured, and colonies were counted.

### Soft agar colony formation

Anchorage-independent growth was evaluated by the soft agar colony formation assay. Briefly, a bottom layer of 0.6% agar (in complete medium) was prepared in 12-well plates. Cells (1000 per well) were suspended in 0.35% agar and plated on top of the solidified base layer. After solidification, plates were incubated at 37 °C in a humidified incubator with 5% CO_2_ for 14 days. Colonies were then fixed with 4% paraformaldehyde and stained with 0.1% crystal violet for 30 min. Images were captured under a light microscope, and colonies larger than 50 μm were counted using ImageJ software.

### HYW preparation, components analysis, and network pharmacology analysis

The preparation of HYW was identical to that described in our previous study (18), including the raw material source, extraction conditions, and final concentration, to ensure methodological consistency and comparability between studies (**Supplementary Table S1**). The chemical composition and active ingredient screening of HYW in the present work were entirely based on the previously reported UHPLC-Q-Orbitrap-HRMS profiling and ADME evaluation (19). Briefly, in the referenced study, UHPLC-Q-Orbitrap-HRMS analysis was performed on an UltiMate 3000 RS UHPLC system coupled with a Q Exactive Orbitrap mass spectrometer (Thermo Fisher Scientific, USA) equipped with a heated electrospray ionization source, using a Waters ACQUITY UPLC HSS T3 C18 column (2.1 × 100 mm, 1.8 μm) at 30 °C. Chromatographic separation employed 0.1% formic acid–water and 0.1% formic acid–acetonitrile as the mobile phases at a flow rate of 0.2 mL/min, and MS data were acquired in both positive and negative ion modes over an m/z range of 100–1500. Compound identification and ADME-based screening followed the same criteria, with active ingredients defined using SwissADME and TCMSP parameters (oral bioavailability OB ≥30% and drug likeness≥0.18). The combined use of in silico ADME evaluation and TCMSP-based screening facilitates the prioritization of bioavailable and drug-like compounds from complex herbal formulations, thereby improving the biological relevance and reliability of subsequent network pharmacology analysis. Network pharmacology analysis and functional enrichment were conducted using publicly available databases and R packages, following previously reported methods (19).

### Flow cytometric analysis

Cell death was determined using Annexin V–FITC/PI staining. After treatment, cells were collected, washed with PBS, and resuspended in binding buffer. Annexin V–FITC and PI (Annexin V–FITC/PI Apoptosis Detection Kit, Beyotime, Shanghai, China) were added and incubated for 15 min at room temperature in the dark. Samples were analyzed using a flow cytometer, and data were processed with FlowJo software.

For the detection of cell-surface calreticulin (CALR) exposure, cells were collected following the indicated treatments and washed with PBS. Cells were incubated with a rabbit anti-calreticulin antibody (Abcam, ab2907; 1:1000) for 60 min, washed, and subsequently incubated with an Alexa Fluor 488-conjugated goat anti-rabbit IgG secondary antibody (Invitrogen, No. A-11008; 1 μg/ml) for 30 min. After washing, cells were analyzed by flow cytometry, and cell-surface CALR exposure was quantified as mean fluorescence intensity (MFI) using FlowJo software.

### Fluorescence microscopy

Intracellular reactive oxygen species (ROS) levels were detected using a DCFH-DA probe (Beyotime, Shanghai, China) according to the manufacturer’s protocol. After treatment, cells were incubated with 10 μM DCFH-DA at 37 °C for 30 min, washed three times with serum-free medium, and immediately observed under a fluorescence microscope (Leica, Germany). The intensity of green fluorescence reflected the intracellular ROS level.

Mitochondrial membrane potential was examined using the JC-1 staining kit (Beyotime, Shanghai, China). Cells were incubated with JC-1 working solution for 20 min at 37 °C, washed twice with JC-1 buffer, and visualized under red and green channels. The ratio of green (JC-1 monomers) to red (JC-1 aggregates) fluorescence was used to evaluate mitochondrial membrane depolarization.

Labile Fe²^+^ was detected using 1 μM FerroOrange (Dojindo, Kumamoto, Japan). After 30 min incubation at 37 °C, cells were gently washed with HBSS and imaged under the orange-red fluorescence channel. The fluorescence intensity reflected the level of intracellular ferrous iron.

### Quantification of total intracellular iron

Total intracellular iron levels were measured using an Iron Assay Kit (Abcam, Cambridge, UK) according to the manufacturer’s instructions. Briefly, cells were collected, washed with cold PBS, and lysed in iron assay buffer. The supernatant was obtained by centrifugation, and the total iron content was quantified colorimetrically at 593 nm using a microplate reader (Bio-Rad, USA). The results were normalized to protein concentration and expressed as relative iron levels compared with the control group.

### Measurement of cellular GSH to GSSG ratio

The cellular reduced and oxidized glutathione levels were determined using a GSH/GSSG Ratio Detection Kit (Beyotime, Shanghai, China) following the manufacturer’s protocol. After treatment, cells were harvested and lysed in the provided buffer containing protein-removal reagent. The supernatant was collected by centrifugation, and total glutathione (GSH + GSSG) and oxidized glutathione (GSSG) were measured colorimetrically at 412 nm using a microplate reader (Bio-Rad, USA). The reduced glutathione (GSH) content was calculated by subtracting GSSG from total glutathione, and the GSH/GSSG ratio was used to evaluate the cellular redox status.

### Measurement of extracellular ATP and LDH release

Extracellular ATP levels were measured using an ATP Assay Kit (Beyotime, Shanghai, China) according to the manufacturer’s instructions. Cell culture supernatants were collected and centrifuged to remove cellular debris. ATP levels were determined using a luciferase-based luminescence assay with a microplate reader and normalized to the control group.

LDH release was measured using an LDH Cytotoxicity Assay Kit (Beyotime, Shanghai, China) according to the manufacturer’s instructions. Cell-free culture supernatants were incubated with the supplied detection reagent, and absorbance was measured at 490 nm using a microplate reader. LDH release was calculated relative to the spontaneous and maximum LDH release controls and expressed as a percentage.

### Determination of malondialdehyde levels

Malondialdehyde (MDA) levels were measured using a Lipid Peroxidation (MDA) Assay Kit (Beyotime, Shanghai, China) according to the manufacturer’s instructions. After treatment, cells were harvested, lysed in MDA lysis buffer, and centrifuged to obtain the supernatant. The MDA content was determined based on the reaction of MDA with thiobarbituric acid (TBA) to form a colored adduct, which was measured colorimetrically at 532 nm. The results were normalized to protein concentration and expressed as relative MDA levels compared with the control group.

### Western blot analysis

Cells or tissue samples were lysed in RIPA buffer (Beyotime, Shanghai, China) supplemented with protease and phosphatase inhibitors. Protein concentrations were determined using the BCA Protein Assay Kit (Thermo Fisher Scientific, USA). Equal amounts of protein (20–40 μg) were separated by SDS–PAGE and transferred onto PVDF membranes (Millipore, USA). The membranes were blocked with 5% non-fat milk for 1h at room temperature and incubated overnight at 4 °C with primary antibodies against the indicated targets. After washing, membranes were incubated with HRP-conjugated secondary antibodies for 1 h at room temperature. Protein bands were visualized using an enhanced chemiluminescence (ECL) detection system (Bio-Rad, USA), and relative protein levels were quantified using ImageJ software.

### Enzyme-linked immunosorbent assay

ELISA was performed as previously described (24), with minor modifications. Tumor tissues were homogenized in ice-cold PBS or an appropriate extraction buffer and centrifuged to remove tissue debris. The resulting supernatants were collected for analysis. HMGB1, CXCL9, and CXCL10 levels were quantified using ELISA kits according to the manufacturers’ instructions. Absorbance was measured using a microplate reader, and concentrations were calculated from the corresponding standard curves. The measured amounts were normalized to the corresponding tumor volume and expressed as pg/mm³.

### Quantitative real time PCR

Total RNA was extracted using TRIzol reagent (Invitrogen, USA), and 1 μg of RNA was reverse-transcribed into cDNA using a reverse transcription kit (Takara, Japan). Quantitative PCR was conducted with SYBR Green Master Mix (Takara, Japan) on a QuantStudio 6 Flex Real-Time PCR System (Applied Biosystems, USA). Relative gene expression levels were calculated using the 2^−^ΔΔCt method and normalized to GAPDH. Primer sequences are listed in **Supplementary Table S2**.

### Cystine uptake assay

Cystine uptake was determined using a Cystine Assay Kit (Elabscience, Wuhan, China) according to the manufacturer’s instructions. Cells were seeded in 6-well plates and incubated with cystine-free medium for 1 h to deplete intracellular cystine. The medium was then replaced with fresh medium containing 200 μM cystine, and cells were incubated for 30 min at 37 °C. After washing with cold PBS, cells were lysed, and intracellular cystine levels were quantified colorimetrically at 560 nm using a microplate reader (Bio-Rad, USA). The results were normalized to protein concentration.

### Animal studies

All animal experiments were conducted in accordance with the Guide for the Care and Use of Laboratory Animals, 8th edition (National Research Council, 2011), and were approved by the Animal Ethics Committee of Shaoxing People’s Hospital (2024Z014). At the experimental endpoint, mice were euthanized by gradual-fill carbon dioxide (CO_2_) inhalation in a non-prefilled chamber at a displacement rate of 30% of the chamber volume per minute, in accordance with institutional animal care and use guidelines.

To evaluate the antitumor efficacy of HYW *in vivo*, six-week-old female C57BL/6 mice were subcutaneously inoculated with 2 × 10^6^ mEC25-CDDP cells in the right flank. On day 5 after tumor inoculation, mice were randomly assigned to three groups: vehicle control, HYW low-dose (200 mg/kg), and HYW high-dose (400 mg/kg). HYW was administered by oral gavage (i.g.) once daily from day 5 until the experimental endpoint on day 25. Control mice received an equivalent volume of vehicle using the same administration schedule. Body weight and tumor size were measured every three days.

To assess whether HYW enhances the antitumor activity of anti-PD-1 therapy, mice were randomly assigned to four groups: control, HYW alone, anti-PD-1 alone, and HYW plus anti-PD-1. HYW (200 mg/kg) was administered by oral gavage once daily during the treatment period. Anti-PD-1 antibody (100 μg/mouse/dose) or the matched isotype control was administered intraperitoneally at the indicated time points for a total of three doses. Tumor size and body weight were recorded every five days, and mice were euthanized at the experimental endpoint.

### Statistical analysis

Data are presented as mean ± SD, except for tumor growth curves, which are presented as mean ± SEM. Statistical analyses were performed using GraphPad Prism 8.0. Comparisons between two groups were performed using an unpaired two-tailed Student’s t-test. Comparisons among three or more groups were performed using one-way ANOVA followed by Tukey’s multiple-comparison test. *In vitro* time-course data were analyzed using two-way ANOVA. A P value <0.05 was considered statistically significant. *P < 0.05, **P < 0.01, and ***P < 0.001; ns, not significant.

## Results

### HYW suppresses the growth of cisplatin-resistant ESCC cells

Cisplatin-resistant ESCC sublines were generated through a stepwise pulse exposure to increasing concentrations of cisplatin using both human and murine ESCC cell lines, KYSE150 and mEC25 (**Figure 1A**). The established KYSE150/CDDP and mEC25/CDDP cells exhibited markedly increased IC50 values for cisplatin compared with their parental counterparts, confirming the successful establishment of cisplatin-resistant ESCC cell models. Moreover, IC50 analysis across different passages showed that both KYSE150/CDDP and mEC25/CDDP cells maintained a relatively stable cisplatin-resistant phenotype from P3 to P10. Therefore, P5 cells, which displayed a robust resistant phenotype while minimizing potential passage-associated phenotypic drift, were selected for all subsequent experiments (**Figure 1B**; **Supplementary Figure S1A**).

**Figure 1:**
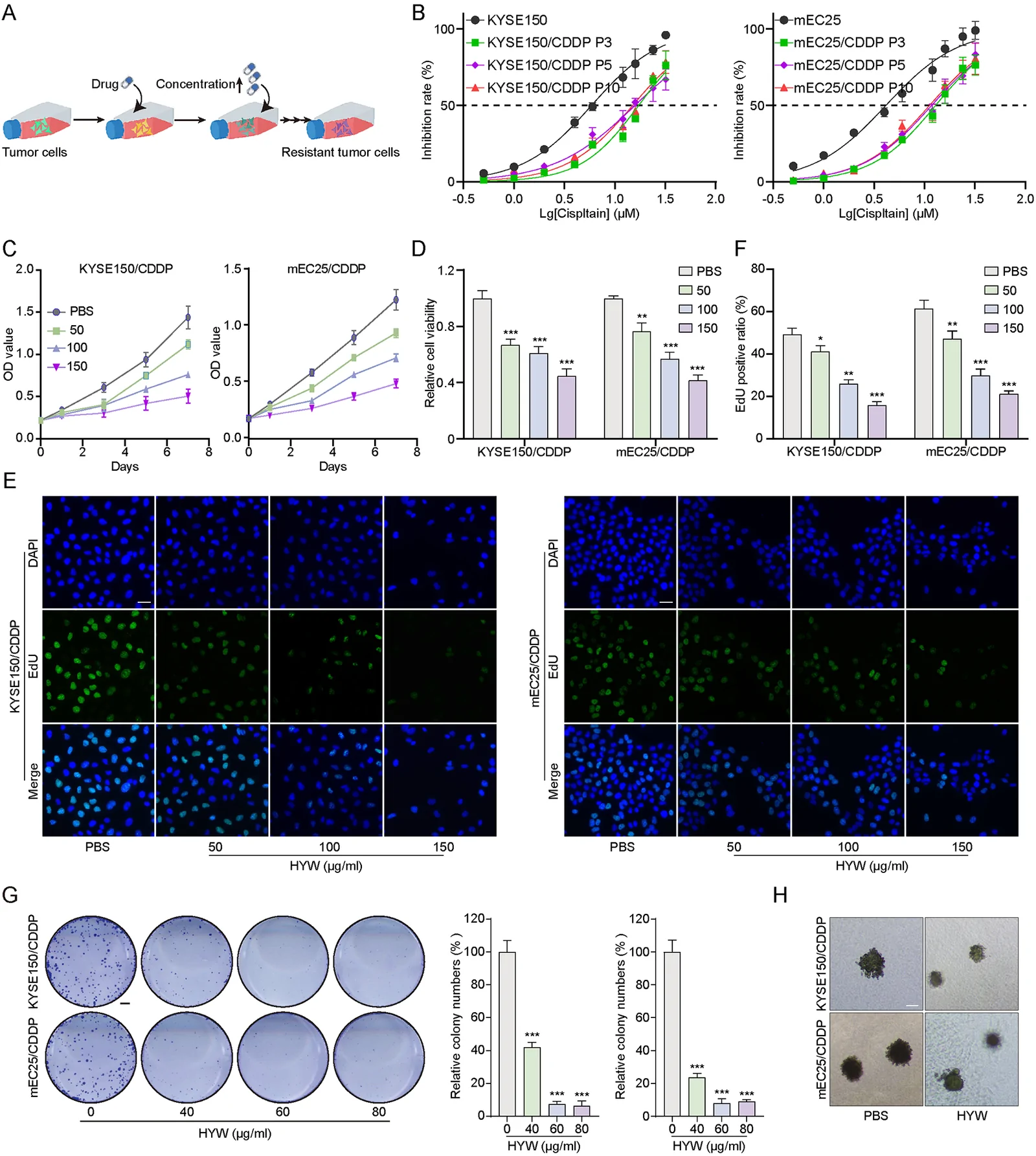
Establishment of cisplatin-resistant ESCC cell lines and effects of HYW on resistant cells. **(A)** Generation of cisplatin-resistant ESCC sublines. **(B)** Cisplatin dose-response curves of parental KYSE150 and mEC25 cells and their cisplatin-resistant derivatives after 3, 5, or 10 drug-free passages (KYSE150/CDDP P3, P5, and P10; mEC25/CDDP P3, P5, and P10). **(C, D)** Effects of HYW on cell growth in KYSE150/CDDP and mEC25/CDDP cells. **(E, F)** EdU incorporation in KYSE150/CDDP and mEC25/CDDP cells treated with HYW. **(G)** Colony formation of KYSE150/CDDP and mEC25/CDDP cells following HYW treatment. **(H)** Soft agar colony formation by KYSE150/CDDP and mEC25/CDDP cells treated with PBS or HYW (40 μg/ml) for 14 days. Data are presented as mean ± SD from three independent biological replicates. *P < 0.05, P** < 0.01, P*** < 0.001.

To evaluate the antitumor potential of HYW, KYSE150-CDDP and mEC25-CDDP cells were treated with increasing concentrations of HYW for varying durations. HYW markedly reduced cell viability in a time- and dose-dependent manner (**Figures 1C, D**).

Notably, HYW also exhibited significant antiproliferative activity against the corresponding parental KYSE150 and mEC25 cells (**Supplementary Figure S1B**). EdU incorporation assays further revealed a pronounced decrease in EdU-positive cells following HYW treatment, indicating inhibition of DNA synthesis and proliferative activity (**Figures 1E, F**). Long-term colony-formation assays showed that HYW significantly suppressed the clonogenic survival of resistant cells (**Figure 1G**). Moreover, HYW markedly impaired the anchorage-independent growth of KYSE150/CDDP and mEC25/CDDP cells, as evidenced by a significant reduction in colony size (**Figure 1H**; **Supplementary Figure S1C**). Collectively, these findings demonstrate that HYW retains substantial antitumor activity in cisplatin-resistant ESCC cells *in vitro*.

### HYW compromises the ferroptotic defense system in cisplatin-resistant ESCC cells

To determine whether HYW disrupts the ferroptotic defense system in cisplatin-resistant ESCC cells, we first examined intracellular redox status and mitochondrial function. To determine whether redox imbalance was involved in the cellular response, intracellular ROS levels and mitochondrial membrane potential were examined after treatment. A marked accumulation of ROS was observed (**Figure 2A**), and JC-1 staining revealed a pronounced loss of mitochondrial membrane potential, indicating mitochondrial depolarization (**Figures 2B, C**). As redox disequilibrium is a key upstream event in ferroptosis, ferroptosis-related biochemical indicators were further analyzed. FerroOrange staining revealed a significant increase in intracellular labile Fe²^+^, while colorimetric analysis showed a concomitant increase in total intracellular iron following HYW treatment (**Figures 2D, E**). Meanwhile, the cellular GSH/GSSG ratio was reduced, and malondialdehyde (MDA) levels were elevated, reflecting enhanced oxidative stress and lipid peroxidation (**Figures 2F, G**). Western blot analysis revealed a concentration-dependent decrease in GPX4 expression, suggesting a weakened ferroptotic defense (**Figure 2H**). To further determine whether HYW-induced ferroptosis was accompanied by immunogenic cell death (ICD), we examined several canonical damage-associated molecular patterns (DAMPs). HYW treatment markedly increased extracellular ATP release in both KYSE150/CDDP and mEC25/CDDP cells in a concentration-dependent manner (**Figure 2I**). Consistently, HMGB1 release into the culture supernatant was also progressively elevated following HYW treatment (**Figure 2J**). Flow cytometric analysis further showed enhanced cell-surface calreticulin (CALR) exposure with increasing concentrations of HYW (**Figure 2K**; **Supplementary Figure S1D**). Collectively, these findings indicate that HYW induces a ferroptotic phenotype characterized by increased ROS and iron accumulation, enhanced lipid peroxidation, and reduced GPX4 expression, accompanied by the release and surface exposure of ICD-associated DAMPs in cisplatin-resistant ESCC cells.

**Figure 2:**
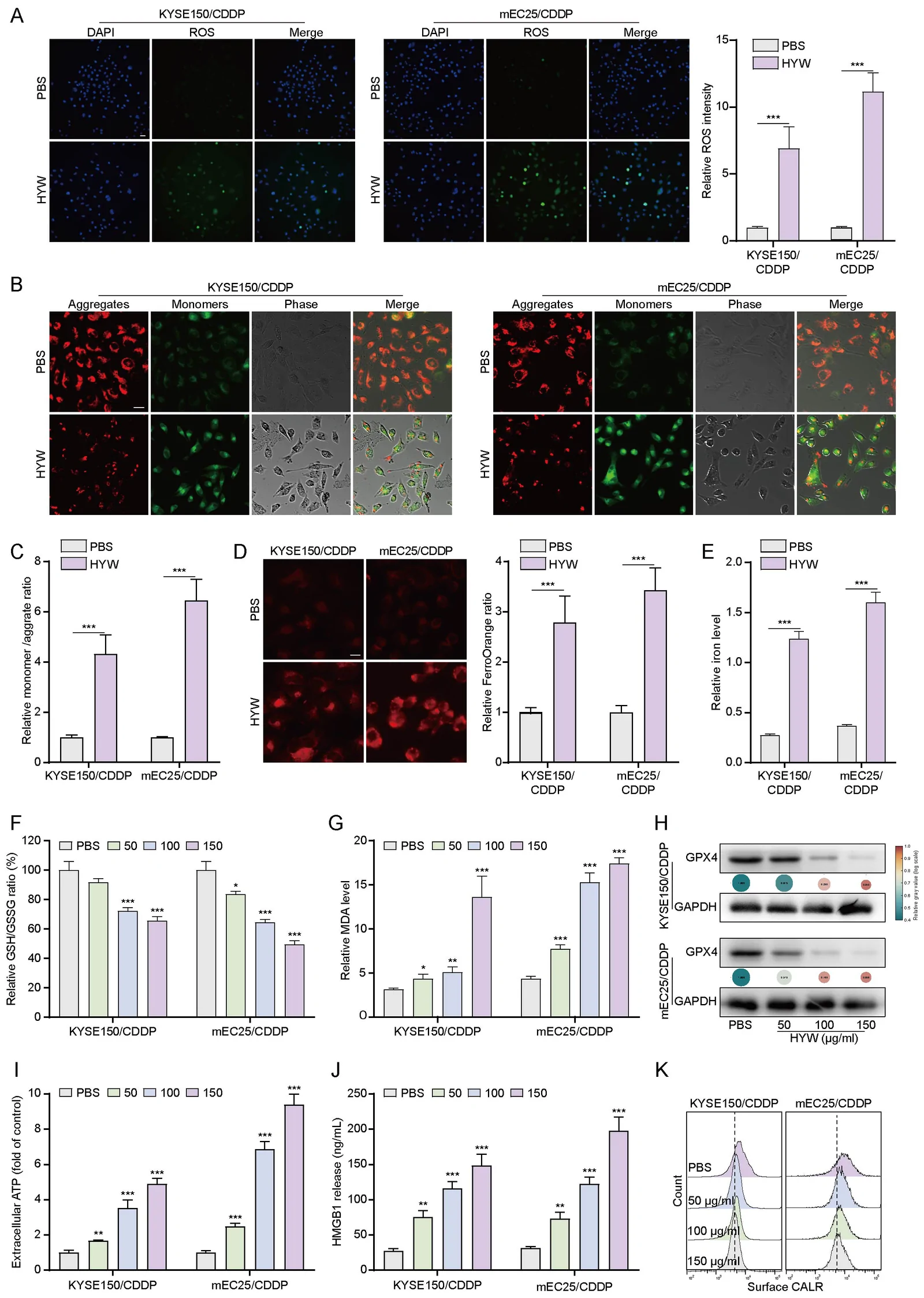
HYW induces ferroptosis-associated redox imbalance and ICD-related DAMP release in cisplatin-resistant ESCC cells. **(A)** Intracellular reactive oxygen species (ROS) levels in KYSE150/CDDP and mEC25/CDDP cells treated with PBS or HYW (100 μg/ml) for 48 h. **(B, C)** HYW-induced changes in mitochondrial membrane potential (ΔΨm) in KYSE150/CDDP and mEC25/CDDP cells treated with PBS or HYW (100 μg/ml) for 48 h, as assessed by JC-1 staining. **(B)** Representative JC-1 fluorescence images. **(C)** Quantification of the monomer-to-aggregate fluorescence ratio. **(D)** Representative FerroOrange fluorescence images showing intracellular labile Fe²^+^ accumulation following treatment with PBS or HYW (100 μg/ml) for 48 h. **(E)** Total intracellular iron levels in KYSE150/CDDP and mEC25/CDDP cells following the same treatment. **(F)** Relative GSH/GSSG ratios in KYSE150/CDDP and mEC25/CDDP cells treated with PBS or HYW for 48 h. **(G)** Relative malondialdehyde (MDA) levels, indicating lipid peroxidation, under the same treatment conditions. **(H)** Expression of the ferroptosis-related protein GPX4. **(I-K)** Assessment of ICD-associated danger signals in KYSE150/CDDP and mEC25/CDDP cells following treatment with PBS or HYW for 48 h. **(I)** Extracellular ATP release. **(J)** HMGB1 release. **(K)** Surface CALR exposure. Data are presented as mean ± SD from three independent biological replicates. *P < 0.05, P** < 0.01, P*** < 0.001.

### Ferroptosis inhibition alleviates HYW-induced cytotoxicity in cisplatin-resistant ESCC cells

To further characterize HYW-induced cell death, different cell death inhibitors were applied. Ferrostatin-1 produced the strongest rescue effect, while Z-VAD-FMK showed a moderate protective effect, and their combination further restored cell viability (**Figure 3A**). These results indicate that both ferroptosis and apoptosis contribute to HYW-induced cell death, with ferroptosis playing the predominant role (**Figure 3A**). Consistent with these findings, HYW markedly suppressed colony formation in both KYSE150/CDDP and mEC25/CDDP cells, whereas ferrostatin-1 partially reversed this inhibitory effect (**Figure 3B**), further supporting a major contribution of ferroptosis to HYW-induced growth inhibition. EdU incorporation assays further showed that in the presence of Fer-1, the inhibitory effect of HYW on cell proliferation was significantly reduced (**Figures 3C, D**). Flow cytometric analysis of Annexin V/PI staining indicated that HYW markedly increased cell death, whereas co-treatment with Fer-1 significantly decreased the death ratio (**Figure 3E**). Consistent with these observations, Fer-1 restored redox balance, as reflected by an increased GSH/GSSG ratio, reduced intracellular iron accumulation, and lower MDA levels compared with HYW treatment alone (**Figures 3F–H**). Collectively, these results demonstrate that the cytotoxic and oxidative responses induced by HYW are largely dependent on ferroptosis.

**Figure 3:**
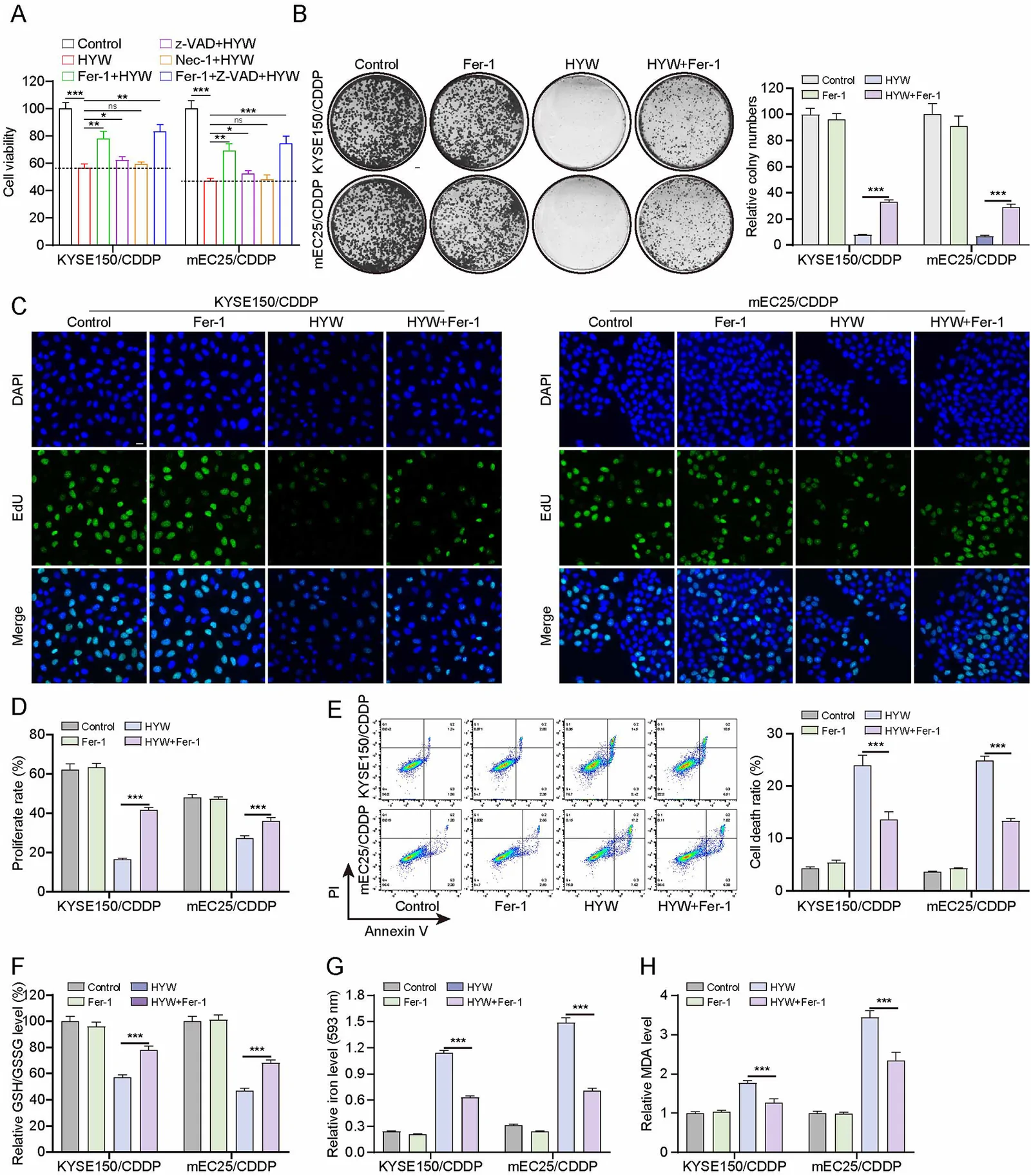
Ferroptosis contributes to HYW-induced cell death in cisplatin-resistant ESCC cells. **(A)** Cell viability of KYSE150/CDDP and mEC25/CDDP cells treated with HYW (100 μg/ml) alone or in combination with ferrostatin-1 (Fer-1, 1 μM), Z-VAD-FMK (Z-VAD, 50 μM), or necrostatin-1 (Nec-1, 2 μM) for 48 h was determined using the CCK-8 assay. **(B)** Colony formation in KYSE150/CDDP and mEC25/CDDP cells treated with HYW (60 μg/ml) in the presence or absence of Fer-1 (1 μM). **(C)** Representative fluorescence images of EdU incorporation assays in cells treated with HYW (100 μg/ml) with or without Fer-1 (1 μM) for 48 h. EdU-positive cells are shown in green and nuclei were counterstained with DAPI (blue). **(D)** Quantification of the EdU-positive proliferation rate. **(E)** Annexin V/PI staining and quantitative analysis of cell death in KYSE150/CDDP and mEC25/CDDP cells treated with HYW (100 μg/ml) with or without Fer-1 (1 μM) for 48 h. **(F–H)** Relative GSH/GSSG levels **(F)**, intracellular iron levels **(G)**, and relative MDA levels **(H)** in cells treated with HYW (100 μg/ml) with or without Fer-1 (1 μM) for 48 h. Data are presented as mean ± SD from three independent biological replicates. *P<0.05, **P < 0.01, ***P < 0.001; ns, not significant.

### Identification of candidate bioactive components and potential therapeutic targets of HYW in ESCC

To identify the pharmacologically active constituents responsible for the antitumor effects of HYW in cisplatin-resistant ESCC, we collected and analyzed 39 reported bioactive compounds that had been previously characterized by UHPLC-Q-Orbitrap-HRMS chemical profiling (19) and subjected them to ADME-based pharmacokinetic screening (**Figure 4A**; **Supplementary Table S3**). According to their chemical structures, these compounds were mainly categorized into alkaloids, flavonoids, terpenoids, and phenylpropanoids (**Figure 4B**). The toxicological characteristics of these phytochemicals were evaluated using the Protox II web server, and most compounds exhibited only two active toxicity endpoints. Among them, L-threonine showed the highest LD_50_ value of 12,680 mg/kg (**Figures 4A–C**). Target prediction and disease association analyses identified 172 overlapping targets, representing 72.3% of the potential therapeutic targets related to ESCC (**Figure 4D**; **Supplementary Table S4**). A compound-target interaction network was subsequently constructed, revealing extensive connections between multiple HYW constituents and ESCC-associated targets (**Figure 4E**). Degree-based network analysis identified quercetin, daidzein, epicatechin, calycosin, medicarpin, morin, oxypeucedanin hydrate, palmatine, allocryptopine, and corydaline as the top-ranked bioactive components (**Supplementary Figure S2A**). To ensure quality control, experimental reproducibility, and a reliable chemical basis for mechanistic interpretation and potential clinical translation, the contents of these key bioactive constituents in HYW were further quantified. Quantitative analysis revealed that allocryptopine was the most abundant compound, with a content of 360.01 mg/kg in HYW (**Supplementary Figure S2B**). To experimentally validate the cellular functions of the core constituents predicted by network pharmacology, the five highest-degree compounds—quercetin, daidzein, epicatechin, calycosin, and medicarpin—were individually evaluated in KYSE150/CDDP and mEC25/CDDP cells. Quercetin, daidzein, epicatechin, and calycosin significantly reduced cell viability, whereas medicarpin exerted only a limited effect (**Supplementary Figures S2C–G**). Importantly, ferrostatin-1 partially rescued the cytotoxicity induced by quercetin, epicatechin, and daidzein, indicating that ferroptosis contributes to the antitumor activity of these core constituents. Consistent with this, quercetin, daidzein, and epicatechin produced prominent alterations in the GSH/GSSG balance and increased MDA accumulation (**Supplementary Figures S2H, I**). These data provide a systems-level view of the chemical diversity and multi-target regulatory potential of HYW in the context of ESCC.

**Figure 4:**
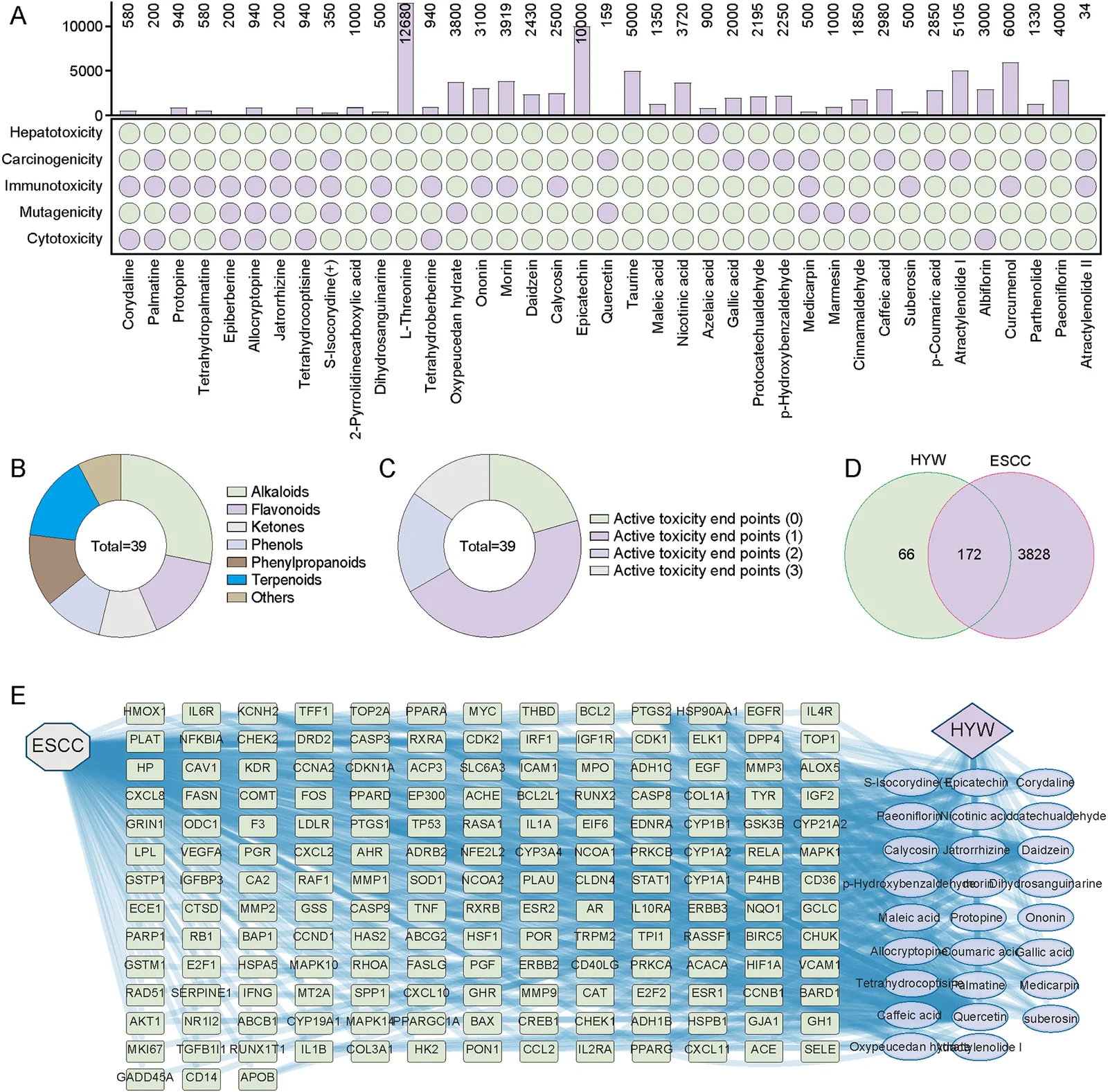
Identification of candidate bioactive components and potential therapeutic targets of HYW. **(A)** Predicted toxicity profiles of bioactive components. **(B)** Chemical classification of HYW phytochemicals. **(C)** Summary of predicted toxicity endpoints. **(D)** Overlap between HYW-related targets and ESCC-associated genes. **(E)** Compound–target interaction network.

### Network-based identification of core targets and signaling pathways underlying HYW action in ESCC

Based on the potential therapeutic targets identified above, a PPI network was constructed to elucidate the core regulatory proteins mediating the effects of HYW on ESCC (**Figures 5A, B**). The resulting network comprised 172 nodes and 7060 edges, indicating extensive interactions among the predicted targets. Topological analysis revealed that several genes, including AKT1, P53, TNF, EGFR, IL6, STAT3, BCL2L1, MAPK1, and HSP90AA1, occupied central positions in the network with high degree values, suggesting their importance as potential key nodes (**Figures 5B, C**). To further clarify the biological significance of these targets, GO and KEGG enrichment analyses were performed. GO enrichment showed that these genes were mainly localized to the external side of the plasma membrane, membrane raft, and vesicle lumen (**Figure 5D**). Notably, biological process enrichment revealed prominent associations with responses to oxidative stress, hypoxia, decreased oxygen levels, and related redox stress processes (**Figure 5E**), which are consistent with the oxidative stress and lipid peroxidation features characteristic of ferroptosis. Functional enrichment indicated strong associations with DNA-binding transcription factor binding and ligand-mediated signaling activity (**Figure 5F**). KEGG analysis revealed enrichment of multiple cancer-related pathways, including the PI3K–AKT and p53 signaling pathways (**Figure 5G**). To further refine the key regulatory mechanisms, the core targets were extracted and subjected to additional pathway enrichment analysis (**Supplementary Figures S3A, B**). This analysis again highlighted the PI3K-AKT and p53 signaling pathways, with AKT1 and TP53 representing prominent core nodes. Collectively, these network pharmacology findings suggest that HYW may exert its therapeutic effects through the coordinated regulation of multiple signaling pathways related to cellular stress response, inflammation, and immune modulation in cisplatin-resistant ESCC.

**Figure 5:**
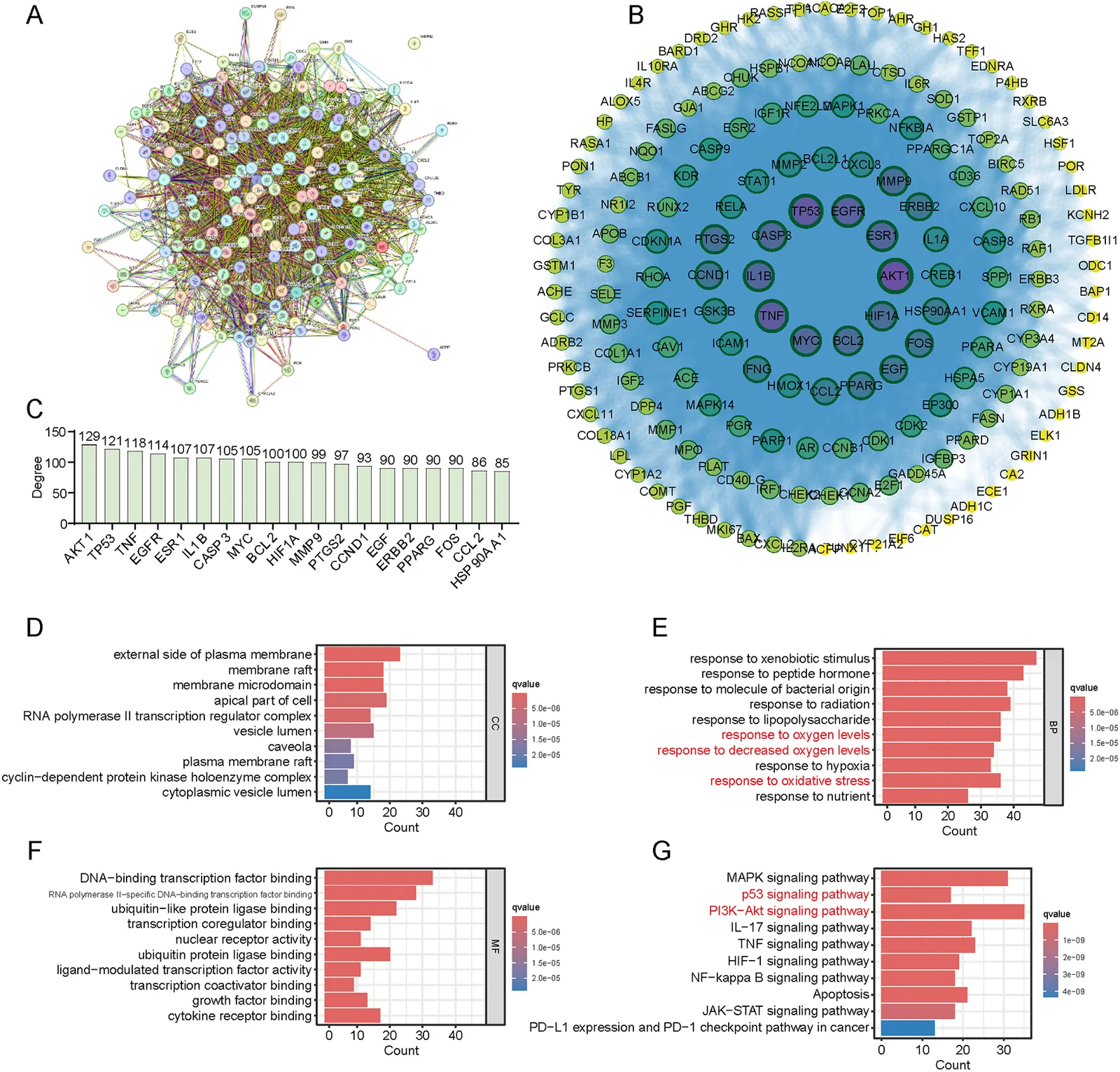
Construction of protein interaction network and enrichment analysis of HYW-associated targets in ESCC. **(A)** PPI network constructed based on predicted therapeutic targets of HYW for ESCC. **(B)** Visualization of the PPI network highlighting central nodes with high degree values. **(C)** Bar graph showing the top 20 hub genes ranked by degree score. **(D–F)** GO enrichment analysis of cellular component **(D)**, biological process **(E)**, and molecular function terms **(F)** for HYW-associated targets in ESCC. **(G)** KEGG pathway enrichment analysis.

### HYW impairs the SLC7A11/GCLC-dependent cystine-glutathione antioxidant system

To further elucidate the molecular mechanism underlying HYW-induced ferroptosis, we integrated the predicted therapeutic targets of HYW with known ferroptosis-related genes and identified their intersection. The overlapping targets were mainly enriched in the glutathione metabolism pathway (**Supplementary Figure S4**; **Figure 6A**). Western blot analysis showed that HYW increased p53 expression while progressively reducing SLC7A11, GCLC, and NRF2 protein levels in both KYSE150/CDDP and mEC25/CDDP cells (**Figure 6B**). Consistently, qPCR analysis revealed decreased expression of SLC7A11, GCLC, and GPX4 following HYW treatment (**Figure 6C**). Notably, NRF2, a central regulator of cellular antioxidant defense and glutathione metabolism, was markedly suppressed by HYW. This change paralleled the reduction in SLC7A11 and GCLC, suggesting that HYW broadly attenuates the antioxidant capacity of cisplatin-resistant ESCC cells. Functionally, these molecular changes were accompanied by a marked decrease in cystine uptake (**Figure 6D**), indicating disruption of the cystine-glutathione antioxidant system. Given the critical role of GCLC in glutathione biosynthesis, we next examined whether restoration of GCLC could counteract the ferroptotic effects of HYW. Successful GCLC overexpression was confirmed at both the mRNA and protein levels (**Figure 6E**). GCLC overexpression partially restored the GSH/GSSG ratio, reduced MDA accumulation, and attenuated HYW-induced growth inhibition (**Figures 6F–H**), indicating that impaired glutathione synthesis contributes to HYW-induced ferroptosis. To further establish the involvement of p53 in this process, cells were treated with the p53 inhibitor PFT-α. Pharmacological inhibition of p53 partially restored SLC7A11 expression and cystine uptake, recovered the GSH/GSSG ratio, and alleviated the reduction in cell viability induced by HYW (**Figures 6I–L**). Collectively, these findings demonstrate that HYW disrupts multiple nodes of the System Xc^−^-glutathione antioxidant pathway, thereby impairing cystine uptake, glutathione metabolism, and redox homeostasis to promote ferroptosis in cisplatin-resistant ESCC cells.

**Figure 6:**
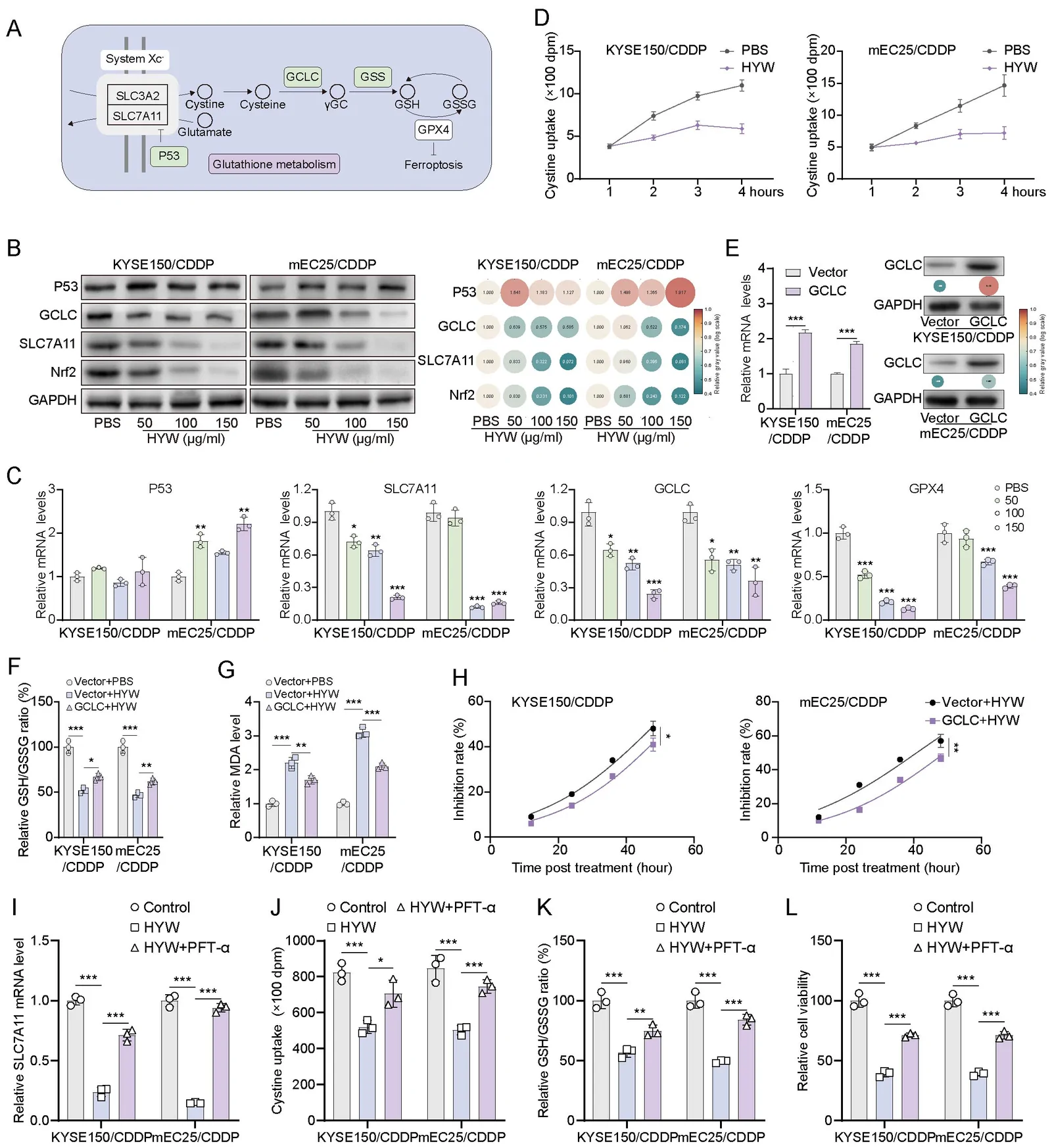
Regulation of the P53-SLC7A11-GCLC signaling axis and glutathione metabolism by HYW in cisplatin-resistant ESCC cells. **(A)** Glutathione metabolism pathway with mapped HYW-related and ferroptosis-associated targets. **(B)** Western blot analysis and densitometric quantification of p53, GCLC, SLC7A11, and NRF2 protein levels. Protein levels were normalized to GAPDH and expressed relative to the PBS-treated control. **(C)** Relative mRNA expression levels of P53, SLC7A11, GCLC, and GPX4 in KYSE150/CDDP and mEC25/CDDP cells following treatment with HYW. **(D)** Cystine uptake in KYSE150/CDDP and mEC25/CDDP cells following treatment with HYW (100 μg/ml) for the indicated times. **(E)** RT-qPCR and western blot validation of GCLC overexpression in KYSE150/CDDP and mEC25/CDDP cells. **(F)** Relative GSH/GSSG ratios in vector control and GCLC-overexpressing KYSE150/CDDP and mEC25/CDDP cells following treatment with HYW (100 μg/ml). **(G)** Relative MDA levels in vector control and GCLC-overexpressing cells following HYW treatment (100 μg/ml). **(H)** Cell growth inhibition rates of vector control and GCLC-overexpressing KYSE150/CDDP and mEC25/CDDP cells following treatment with HYW (100 μg/ml) for the indicated times. **(I)** Relative SLC7A11 mRNA levels in KYSE150/CDDP and mEC25/CDDP cells treated with HYW (100 μg/ml) in the presence or absence of the p53 inhibitor pifithrin-α (PFT-α, 10 μM). **(J)** Cystine uptake in KYSE150/CDDP and mEC25/CDDP cells following treatment with HYW (100 μg/ml) with or without PFT-α (10 μM). **(K)** Relative GSH/GSSG ratios in KYSE150/CDDP and mEC25/CDDP cells under the indicated treatments. **(L)** Relative cell viability of KYSE150/CDDP and mEC25/CDDP cells treated with HYW (100 μg/ml) in the presence or absence of PFT-α (10 μM). Data are presented as mean ± SD from three independent biological replicates. *P < 0.05, **P < 0.01, ***P < 0.001.

### HYW activates autophagy and ferritinophagy via inhibition of the AKT–mTOR pathway in cisplatin-resistant ESCC cells

Given the pronounced oxidative stress induced by HYW and the enrichment of PI3K-AKT signaling identified by network pharmacology, we next investigated whether autophagy-dependent iron mobilization contributes to HYW-induced ferroptosis. HYW treatment markedly increased LC3B puncta in KYSE150/CDDP and mEC25/CDDP cells (**Figure 7A**). Analysis of iron metabolism-related genes further revealed prominent upregulation of NCOA4 accompanied by reduced FTH expression, together with alterations in STEAP3 and TFRC expression (**Figure 7B**). Consistently, western blot analysis showed increased NCOA4 and LC3-II levels and decreased FTH expression following HYW treatment (**Figure 7C**), suggesting enhanced NCOA4-associated ferritinophagy. We next examined the potential upstream signaling mechanism. HYW progressively reduced AKT and mTOR phosphorylation, as reflected by decreased p-AKT/AKT and p-mTOR/mTOR ratios (**Figure 7D**), suggesting suppression of the AKT–mTOR pathway. Functionally, inhibition of autophagy with 3-MA partially attenuated HYW-induced iron accumulation and MDA elevation, reduced LDH release, and restored cell viability (**Figures 7E–H**), supporting the involvement of autophagy in HYW-induced ferroptotic cell death. To further establish the functional role of NCOA4-mediated ferritinophagy, NCOA4 was silenced in HYW-treated cells. NCOA4 knockdown reduced LC3-II accumulation (**Figure 7I**), markedly decreased intracellular iron levels (**Figure 7J**), and partially rescued cell viability (**Figure 7K**). Collectively, these findings support a functional role for NCOA4-associated ferritinophagy in HYW-induced iron mobilization and ferroptotic cell death. The concurrent suppression of AKT–mTOR signaling suggests that this pathway may contribute to HYW-induced ferritinophagy.

**Figure 7:**
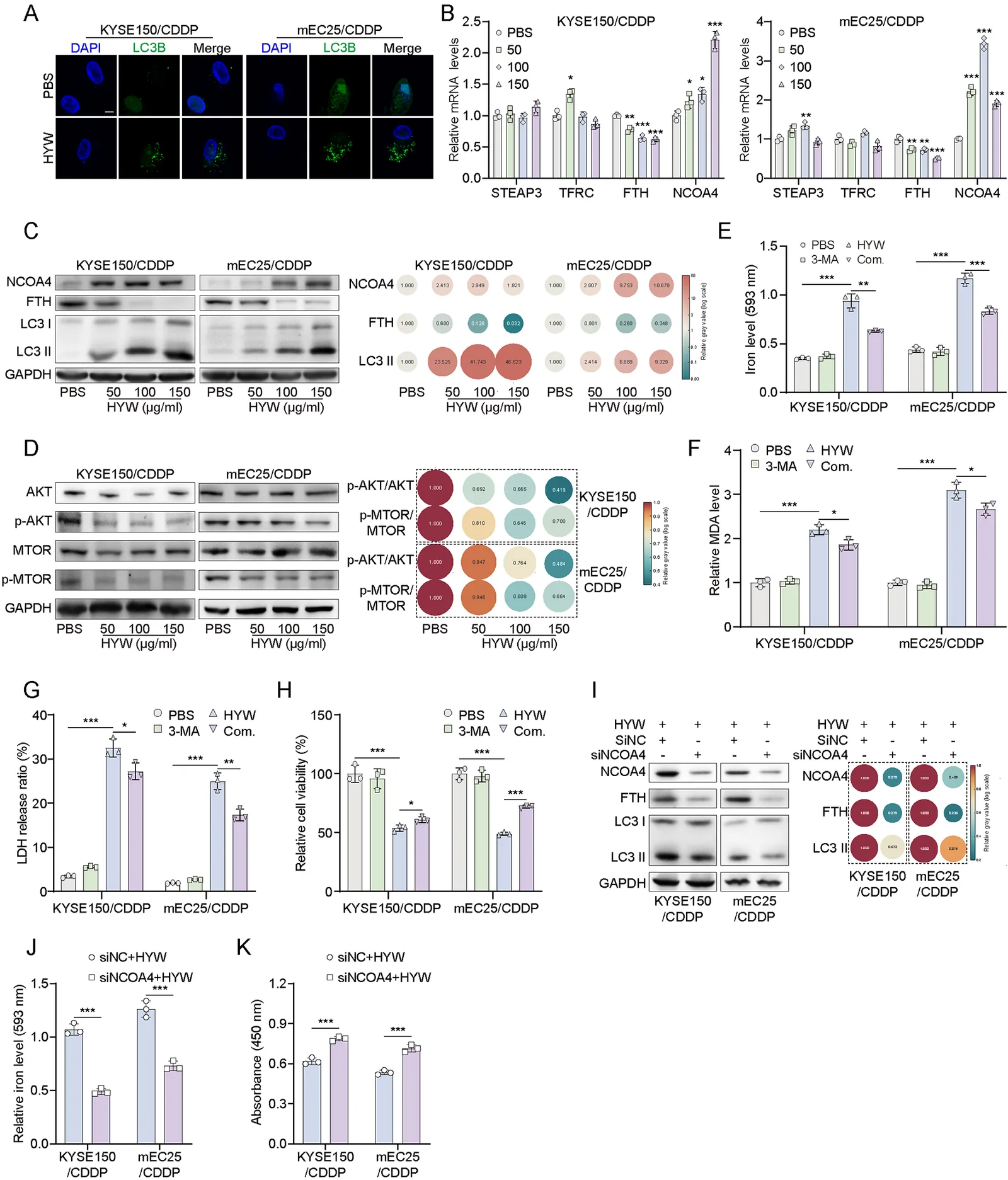
HYW promotes NCOA4-associated ferritinophagy accompanied by suppression of AKT–mTOR signaling in cisplatin-resistant ESCC cells. **(A)** LC3B expression in KYSE150/CDDP and mEC25/CDDP cells following treatment with PBS or HYW (100 μg/ml) for 48 h. **(B)** Relative mRNA levels of STEAP3, TFRC, FTH, and NCOA4 in KYSE150/CDDP and mEC25/CDDP cells treated with HYW for 48 h. **(C)** Western blot analysis of NCOA4, FTH, LC3-I, and LC3-II expression in KYSE150/CDDP and mEC25/CDDP cells treated with increasing concentrations of HYW for 48 h. **(D)** Western blot analysis of AKT, phosphorylated AKT (p-AKT), mTOR, and phosphorylated mTOR (p-mTOR) in KYSE150/CDDP and mEC25/CDDP cells following treatment with increasing concentrations of HYW for 48 h. The relative p-AKT/AKT and p-mTOR/mTOR ratios are shown in the accompanying bubble plots. **(E–H)** Intracellular iron levels **(E)**, relative MDA levels **(F)**, LDH release **(G)**, and relative cell viability **(H)** in KYSE150/CDDP and mEC25/CDDP cells treated with PBS, 3-methyladenine (3-MA, 5 mM), HYW (100 μg/ml), or their combination for 48 h. **(I)** Western blot analysis of NCOA4, FTH, LC3-I, and LC3-II in KYSE150/CDDP and mEC25/CDDP cells treated with HYW (100 μg/ml) after transfection with control siRNA (siNC) or NCOA4-targeting siRNA (siNCOA4). GAPDH was used as the loading control. Relative protein levels are shown in the accompanying bubble plots. **(J)** Intracellular iron levels in HYW-treated (100 μg/ml) KYSE150/CDDP and mEC25/CDDP cells following NCOA4 knockdown. **(K)** Cell viability, assessed by absorbance at 450 nm, in KYSE150/CDDP and mEC25/CDDP cells treated with HYW (100 μg/ml) following transfection with siNC or siNCOA4. Data are presented as mean ± SD from three independent biological replicates. *P < 0.05, **P < 0.01, ***P < 0.001.

### HYW suppresses tumor growth and enhances ferroptosis and immune activation *in vivo*

To further evaluate the therapeutic efficacy of HYW *in vivo*, a cisplatin-resistant ESCC syngeneic tumor model was established in C57BL/6 mice (**Figure 8A**). HYW treatment markedly suppressed tumor growth in a dose-dependent manner, as evidenced by reduced tumor volume and tumor weight compared with the PBS group (**Figures 8B, C**). No obvious changes in body weight or major organ indices were observed during treatment (**Figures 8D, E**). Consistently, serum biochemical analysis after the same treatment period showed no marked alterations in ALT, AST, or BUN levels, although a modest increase in creatinine was observed in the high-dose group (**Figure 8F**), indicating generally acceptable systemic tolerability. Analysis of tumor tissues showed that HYW significantly reduced the GSH/GSSG ratio and increased MDA levels (**Figures 8G, H**), consistent with enhanced oxidative stress and lipid peroxidation *in vivo*. In parallel, HYW treatment increased HMGB1 and ATP release and elevated CXCL9 and CXCL10 levels in tumor tissues (**Figures 8I–L**), indicating induction of an immunogenic stress response. Immunofluorescence analysis further revealed increased TUNEL positivity and reduced GPX4 expression following HYW treatment (**Figure 8M**), supporting the occurrence of ferroptosis-associated cell death *in vivo*. Moreover, HYW reduced PD-L1 expression while increasing CD8α-positive T-cell infiltration (**Figure 8N**; **Supplementary Figure S5**), suggesting remodeling of the tumor immune microenvironment toward enhanced antitumor immunity. To further assess whether HYW could improve the response to immune checkpoint blockade, HYW was combined with anti-PD-1 treatment. The combination produced a greater suppression of tumor growth and a further reduction in tumor weight than either monotherapy, without obvious changes in body weight (**Figures 8O–Q**). Together, these findings demonstrate that HYW suppresses cisplatin-resistant ESCC growth *in vivo* by inducing ferroptosis-associated oxidative stress and immunogenic responses, while promoting a more immune-active tumor microenvironment and enhancing the antitumor efficacy of PD-1 blockade.

**Figure 8:**
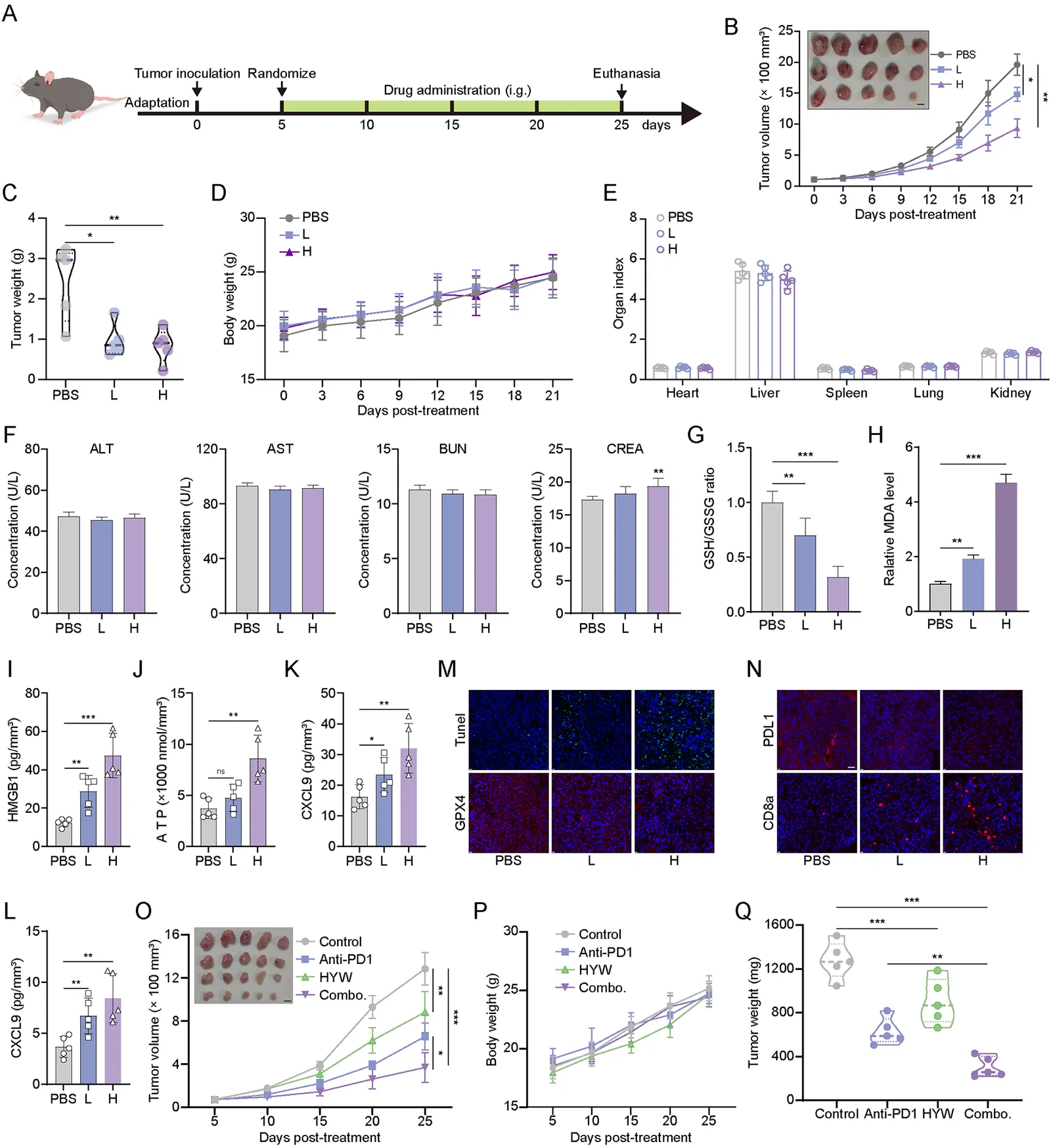
Evaluation of HYW treatment and immune modulation in cisplatin-resistant ESCC syngeneic models. **(A)** Experimental scheme showing the establishment of cisplatin-resistant ESCC syngeneic model and the HYW administration schedule. **(B)** Tumor growth curves and representative excised tumors from mice treated with PBS, low-dose HYW (L, 200 mg/kg), or high-dose HYW (H, 400 mg/kg). **(C)** Tumor weights at the endpoint of treatment. **(D)** Body weight changes during treatment. **(E)** Organ indices of the heart, liver, spleen, lung, and kidney at the endpoint. **(F)** Serum alanine aminotransferase (ALT), aspartate aminotransferase (AST), blood urea nitrogen (BUN), and creatinine (CREA) levels were measured to evaluate systemic toxicity and hepatic and renal function. **(G)** Relative GSH/GSSG ratios and **(H)** relative MDA levels in tumor tissues. **(I–L)** Levels of HMGB1 (**I**), ATP **(J)**, CXCL9 **(K)**, and CXCL10 **(L)** in tumor-associated samples following treatment with PBS or HYW at the indicated doses. **(M)** Representative immunofluorescence images of TUNEL and GPX4 staining in tumor tissues from the indicated treatment groups. **(N)** Representative immunofluorescence images of PD-L1 and CD8α staining in tumor tissues. **(O)** Tumor growth curves and representative excised tumors from mice treated with control, anti-PD-1(100 μg/dose), HYW (200 mg/kg), or the combination of HYW and anti-PD-1. **(P)** Body weight changes during the combination-treatment experiment. **(Q)** Tumor weights at the endpoint of the combination-treatment experiment. Tumor growth curves are presented as mean ± SEM; other quantitative data are presented as mean ± SD. Each mouse represents an independent biological replicate (n = 5 mice per group). *P < 0.05, P < 0.01, **P<0.01, ***P < 0.001; ns, not significant.

## Discussion

Natural products are increasingly recognized for their potential to overcome chemotherapy resistance or increase drug sensitivity by modulating cancer cell survival pathways (25–29). Numerous plant-derived bioactive compounds have resensitized drug-resistant tumors; for example, the flavonoid wogonin selectively kills cisplatin-resistant cancer cells by elevating ROS and downregulating NRF2 antioxidant signaling (30). Moreover, diverse natural substances such as Polyphyllin B (31), Myricetin (32), and Baicalin (33) can directly trigger ferroptotic death in cancer cells, highlighting an untapped reservoir of ferroptosis-inducing therapeutics. In this context, our findings demonstrate that HYW retains antitumor activity in cisplatin-resistant ESCC models and induces ferroptotic cell death through disruption of antioxidant defense and ferritin homeostasis. By coupling these two processes, HYW innovatively forces a breakdown of the cancer cells’ redox defenses: it sparks uncontrolled lipid peroxidation while freeing iron from intracellular ferritin stores, which together drive robust ferroptotic cell death. Notably, ferritinophagy expands the labile iron pool and thereby promotes ferroptosis (34). Together, these changes provide a mechanistic explanation for the ferroptotic response of cisplatin-resistant ESCC cells to HYW treatment.

Cisplatin resistance in ESCC is closely tied to a reprogramming of redox metabolism that enables cancer cells to evade ferroptotic stress. Cisplatin-resistant tumors often exhibit lower baseline ROS and lipid peroxidation levels than their sensitive counterparts (35), reflecting heightened antioxidant capacity and reduced ferroptosis. A key aspect of this redox reprogramming is the upregulation of glutathione biosynthesis and cystine import: for instance, recent work showed that cisplatin-resistant ESCC cells increase expression of the cystine transporter SLC7A11 to bolster glutathione and thwart ferroptosis (36). Consistent with these observations, our findings suggest that HYW compromises glutathione-dependent antioxidant defense through coordinated alterations in cystine uptake and glutathione synthesis. The tumor suppressor P53 is known to inhibit cystine uptake by repressing SLC7A11, thereby sensitizing cells to ferroptosis (37). In parallel, Nrf2 is a major transcriptional regulator of cellular antioxidant defense and supports ferroptosis resistance through the regulation of genes involved in cystine transport, glutathione synthesis, and lipid peroxide detoxification (38). In our study, HYW reduced NRF2 protein expression together with SLC7A11 and GCLC, suggesting that attenuation of NRF2-associated antioxidant signaling may further compromise the glutathione-dependent defense capacity of resistant ESCC cells. In cisplatin resistant cells, HYW lowered SLC7A11 and GCLC, the catalytic subunit of glutamate cysteine ligase required for GSH synthesis, leading to GSH depletion and loss of GPX4 mediated lipid peroxide detoxification. Importantly, HYW engaged the AKT/mTOR/NCOA4 axis to induce ferritinophagy. Active AKT mTOR signaling is a well-known suppressor of autophagy and ferroptosis, and oncogenic activation of this pathway can block NCOA4 mediated ferritinophagy and prevent iron driven ferroptotic cell death (39). By inhibiting AKT/mTOR, HYW unleashes ferritinophagy, causing ferritin degradation and release of iron, which in turn catalyzes toxic lipid peroxidation. This dual action, which impairs GSH based antioxidation while expanding the labile iron pool, establishes a state of ferroptotic vulnerability that overcomes the redox plasticity of cisplatin resistant ESCC cells.

Beyond direct tumoricidal effects, our findings shed light on a coupling between ferroptosis induction and immune remodeling in the tumor microenvironment. Intriguingly, HYW-treated ESCC tumors showed markedly increased CD8^+^ T cell infiltration alongside a decrease in PD-L1 expression on tumor cells. This observation aligns with emerging evidence that links ferroptotic cell death to an enhanced anti-tumor immune response. Ferroptosis is increasingly considered a form of immunogenic cell death: ferroptotic cancer cells release danger-associated molecular patterns (e.g. HMGB1, ATP) that can activate dendritic cells and initiate T-cell priming (34). It is thus plausible that HYW-induced ferroptosis converted the tumor milieu into an immunostimulatory niche, facilitating the recruitment and activation of CD8^+^ cytotoxic T cells. The reduced PD-L1 levels in HYW-treated tumors further suggest a break in immune evasion. Notably, recent work revealed that tumor-intrinsic PD-L1 can itself modulate ferroptosis sensitivity by repressing SLC7A11 and glutathione production (40). These lines of inquiry collectively suggest that both natural products and metabolic pathways can serve as levers for immune regulation (41, 42). Tumors with high PD-L1 expression appear intrinsically more susceptible to ferroptotic death, and HYW may preferentially eliminate this immune evasive population, thereby reducing tumor burden, attenuating a dominant immunosuppressive checkpoint, and enabling deeper infiltration and effector activity of cytotoxic T cells. Together, these data support a model in which HYW induced ferroptosis remodels the tumor microenvironment toward immune activation, lowers PD-L1 expression, enhances CD8^+^ T cell infiltration, and thereby potentiates the antitumor efficacy of anti-PD 1 blockade *in vivo*.

## Conclusions

In summary, this study identifies HYW as a ferroptosis-inducing treatment with antitumor activity in cisplatin-resistant ESCC models. HYW disrupts glutathione-centered antioxidant defense and promotes ferritinophagy-associated iron mobilization, thereby enhancing lipid peroxidation and ferroptotic cell death. *In vivo*, HYW suppresses the growth of cisplatin-resistant ESCC tumors and is associated with reduced PD-L1 expression, increased CD8^+^ T-cell infiltration, and enhanced antitumor activity of PD-1 blockade(**Figure 9**). These findings highlight the potential of HYW as an alternative therapeutic strategy for cisplatin-resistant ESCC and warrant further investigation of its ability to complement existing treatments.

**Figure 9:**
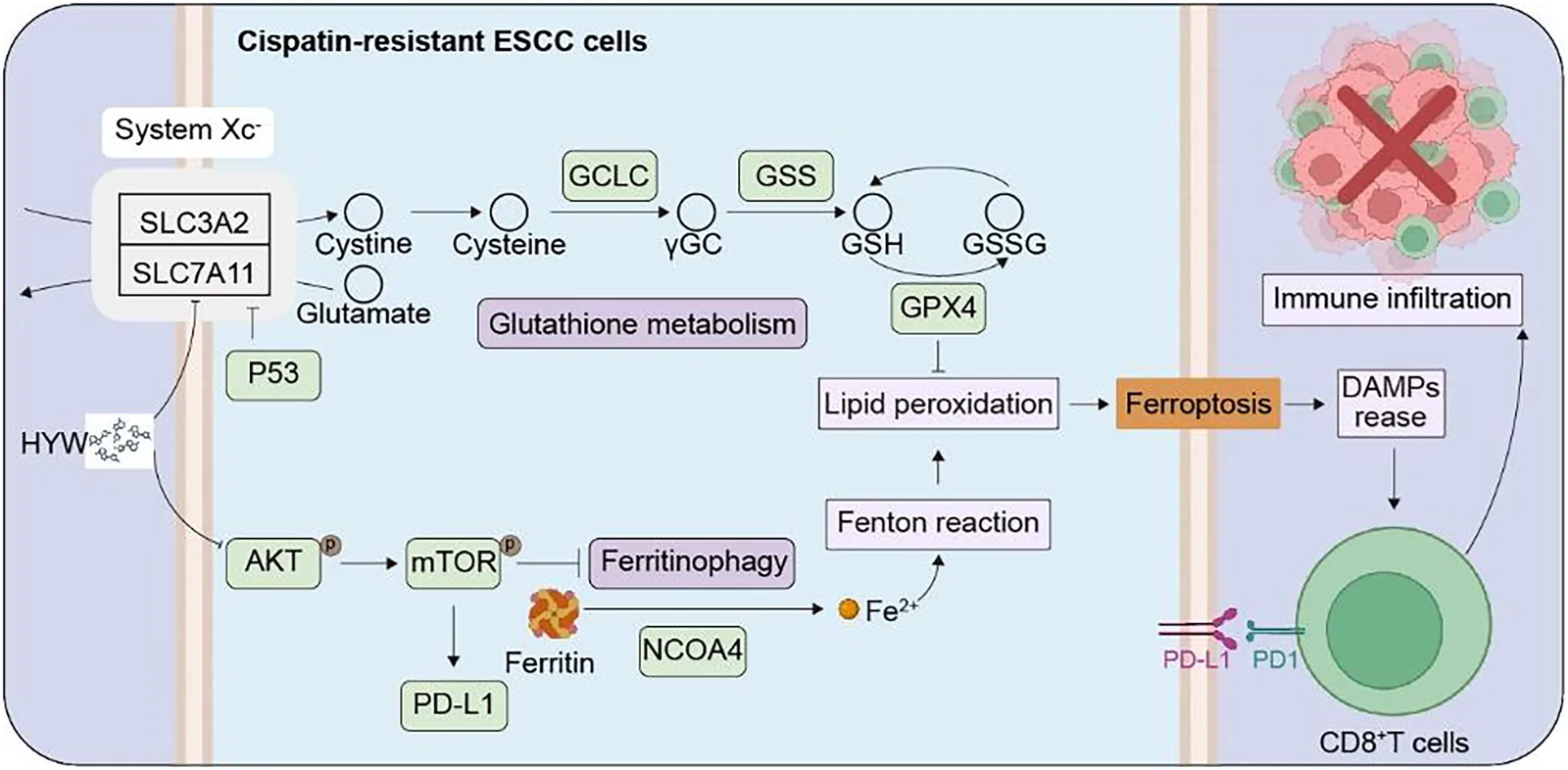
Schematic illustration of the proposed mechanism by which Huayu Wan induces ferroptosis and immune remodeling in cisplatin-resistant esophageal squamous cell carcinoma via glutathione disruption and ferritinophagy activation.

## Data Availability

The datasets presented in this study can be found in online repositories. The names of the repository/repositories and accession number(s) can be found in the article/**Supplementary Material**.

## Supplementary material

Supplementary Figure 1 Comparison of cisplatin sensitivity and HYW response in parental and cisplatin-resistant ESCC cell lines. **(A)** IC_50_ values of cisplatin corresponding to **Figure 1B**, showing the stability of cisplatin resistance in KYSE150/CDDP and mEC25/CDDP cells across passages 3, 5, and 10. **(B)** CCK-8 assay measuring relative cell viability of parental KYSE150 and mEC25 cells treated with different concentrations of HYW (50, 100, and 150 μg/ml) compared with PBS control. **(C)** Quantification of colony diameter corresponding to **Figure 1H** in KYSE150/CDDP and mEC25/CDDP cells treated with PBS or HYW. **(D)** Quantification of cell-surface calreticulin (CALR) expression corresponding to **Figure 2K**, expressed as mean fluorescence intensity (MFI), in KYSE150/CDDP and mEC25/CDDP cells following treatment with HYW at the indicated concentrations (0, 50, 100, and 150 μg/ml). Data are presented as mean ± SD. **P < 0.01, ***P < 0.001.

Supplementary Figure 2 Prioritization and quantitative distribution of key bioactive components in HYW and evaluation of their ferroptosis-related effects. **(A)** Degree ranking of bioactive compounds in the compound–target network. **(B)** Relative content distribution of representative high-degree components in HYW. **(C–G)** Relative cell viability of KYSE150/CDDP and mEC25/CDDP cells treated with quercetin (40 μM; **C**), daidzein (25 μM; **D**), (−)-epicatechin (30 μM; **E**), calycosin (100 μM; **F**), or medicarpin (100 μM; **G**), in the presence or absence of ferrostatin-1 (Fer-1). **(H)** Relative GSH/GSSG ratios in KYSE150/CDDP and mEC25/CDDP cells following treatment with quercetin (40 μM), daidzein (25 μM), (−)-epicatechin (30 μM), calycosin (100 μM), or medicarpin (100 μM). **(I)** Relative MDA levels in KYSE150/CDDP and mEC25/CDDP cells following treatment with the indicated bioactive compounds at the same concentrations. Data are presented as mean ± SD. *P < 0.05, **P < 0.01, ***P < 0.001; ns, not significant.

Supplementary Figure 3 Identification of core targets and pathway enrichment analysis associated with HYW. **(A)** Protein–protein interaction network of the core targets identified from **Figure 5B**. **(B)** Chord diagram showing KEGG pathway enrichment of the core targets.

Supplementary Figure 4 Intersection of predicted HYW targets with the ferroptosis pathway. KEGG ferroptosis map annotated with candidate HYW targets that overlap a curated ferroptosis gene set. Nodes highlighted in red denote overlapping targets.

Supplementary Figure 5 Quantitative analysis of PD-L1 expression and CD8α-positive T-cell infiltration in tumor tissues. **(A)** Relative PD-L1 fluorescence intensity. **(B)** Quantification of CD8α-positive cells per field. Data are presented as mean ± SD. *P < 0.05, **P < 0.01, ***P < 0.001.
