# Supplementary material for: Huayu Wan induces ferroptosis and enhances anti-PD-1 efficacy in cisplatin-resistant esophageal squamous cell carcinoma
Source: Front Immunol. 2026 Oct 27;17:1967436. doi: 10.3389/fimmu.2026.1967436 (PMC13747869; doi:10.3389/fimmu.2026.1967436)
Supplement: Supplementary file 1 [file Table1.docx]

**Table s1**

Botanical and Zoological Composition of the HYW Formulation.

| Latin Name | Chinese Name | Dose (g) | Lot Number |
| --- | --- | --- | --- |
| *Amomum villosum* Lour. | Sharen | 30 | 21,100,297 |
| *Crassostrea gigas* (Thunberg, 1793) | Muli | 90 | 20,120,049 |
| *Hirudo nipponia* Whitman, 1886 | Shuizhi | 10 | 21,100,279 |
| *Tabanus bovinus* Linnaeus, 1758 | Mengchong | 40 | 20,110,004 |
| *Silene vulgaris* (Moench) Garcke | Wangbuliuxing | 60 | 21,100,039 |
| *Prunus persica* (L.) Batsch | Taoren | 90 | 21,100,339 |
| *Paeonia lactiflora* Pall. | Chishao | 150 | 21,040,114 |
| *Astragalus mongholicus* Bunge | Huangqi | 250 | 21,100,328 |
| *Glehnia littoralis* (J.G.Cooper) F.Schmidt | Quanshen | 40 | 21,103,666 |
| *Placenta* *Hominis* | Ziheche | 20 | 21,040,102 |
| *Angelica dahurica* (Hoffm.) Benth. & Hook.f. | Baizhi | 100 | 21,090,163 |
| *Carthamus tinctorius* L. | Honghua | 90 | 21,090,153 |
| *Curcuma aromatica* Salisb. | Yujin | 60 | 21,100,145 |
| *Erica montis-hominis* E.G.H.Oliv. | Erica | 80 | 21,050,031 |
| *Corydalis yanhusuo* W.T.Wang | Yanhusuo | 100 | 21,100,220 |
