# Supplementary material for: Huayu Wan induces ferroptosis and enhances anti-PD-1 efficacy in cisplatin-resistant esophageal squamous cell carcinoma
Source: Front Immunol. 2026 Oct 27;17:1967436. doi: 10.3389/fimmu.2026.1967436 (PMC13747869; doi:10.3389/fimmu.2026.1967436)
Supplement: Supplementary file 2 [file Table2.docx]

**Table s2**. Primers used in this study

| Gene name | Primer name | Annealing Temperature (^◦^C) | Sequence (5′–3′) |
| --- | --- | --- | --- |
| TP53 | TP53-F | 62.4 | CAGCACATGACGGAGGTTGT |
|  | TP53-R | 60.1 | TCATCCAAATACTCCACACGC |
| Trp53 | Trp53-F | 61.4 | CCCCTGTCATCTTTTGTCCCT |
|  | Trp53-R | 60.2 | AGCTGGCAGAATAGCTTATTGAG |
| SLC7A11 | SLC7A11-F | 61.1 | TCTCCAAAGGAGGTTACCTGC |
|  | SLC7A11-R | 60.5 | AGACTCCCCTCAGTAAAGTGAC |
| Slc7a11 | Slc7a11-F | 62.5 | GGCACCGTCATCGGATCAG |
|  | Slc7a11-R | 62.0 | CTCCACAGGCAGACCAGAAAA |
| GCLC | GCLC-F | 62.7 | GGAGGAAACCAAGCGCCAT |
|  | GCLC-R | 61.9 | CTTGACGGCGTGGTAGATGT |
| Gclc | Gclc-F | 62.2 | CTACCACGCAGTCAAGGACC |
|  | Gclc-R | 60.5 | CCTCCATTCAGTAACAACTGGAC |
| GPX4 | GPX4-F | 60.6 | GAGGCAAGACCGAAGTAAACTAC |
|  | GPX4-R | 61.8 | CCGAACTGGTTACACGGGAA |
| Gpx4 | Gpx4-F | 62.1 | TGTGCATCCCGCGATGATT |
|  | Gpx4-R | 60.4 | CCCTGTACTTATCCAGGCAGA |
