# Supplementary material for: Huayu Wan induces ferroptosis and enhances anti-PD-1 efficacy in cisplatin-resistant esophageal squamous cell carcinoma
Source: Front Immunol. 2026 Oct 27;17:1967436. doi: 10.3389/fimmu.2026.1967436 (PMC13747869; doi:10.3389/fimmu.2026.1967436)
Supplement: Supplementary file 3 [file Table3.docx]

**Table s3** Bioactive compounds of Huayuwan.

| NO. | Metabolites | Formula | m/z | RT (min) | Ionization model | Classification | Origin |
| --- | --- | --- | --- | --- | --- | --- | --- |
| 1 | Corydaline | C22H27NO4 | 369.19 | 29.04 | [M+H]+1 | Alkaloids | Corydalis yanhusuo |
| 2 | Palmatine | C21H21NO4 | 351.47 | 29.83 | [M+H]+1 | Alkaloids | Corydalis yanhusuo |
| 3 | Protopine | C_20_H_19_NO_5_ | 353.13 | 27.53 | [M+H]+1 | Alkaloids | Corydalis yanhusuo |
| 4 | Tetrahydropalmatine | C21H25NO4 | 355.18 | 27.95 | [M+H]+1 | Alkaloids | Corydalis yanhusuo |
| 5 | Epiberberine | C20H18NO4 | 332.12 | 30.12 | [M+H]+1 | Alkaloids | Astragalus membranaceus |
| 6 | Allocryptopine | C21H23NO5 | 369.16 | 28.11 | [M+H]+1 | Alkaloids | Corydalis yanhusuo |
| 7 | Jatrorrhizine | C20H19NO4 | 337.13 | 28.24 | [M+H]+1 | Alkaloids | Astragalus membranaceus |
| 8 | Tetrahydrocoptisine | C19H17NO4 | 323.11 | 28.39 | [M+H]+1 | Alkaloids | Corydalis yanhusuo |
| 9 | S-Isocorydine(+) | C20H23NO4 | 341.16 | 26.04 | [M+H]+1 | Alkaloids | Paeonia lactiflora |
| 10 | Tetrahydroberberine | C20H21NO4 | 339.15 | 28.90 | [M+H]+1 | Alkaloids | Corydalis yanhusuo |
| 11 | Dihydrosanguinarine | C20H15NO4 | 333.10 | 45.41 | [M+H]+1 | Alkaloids | Corydalis yanhusuo |
| 12 | L-Threonine | C4H9NO3 | 119.06 | 1.61 | [M+H]+1 | Amino acid | Crassostrea gigas |
| 13 | 2-Pyrrolidinecarboxylic acid | C5H9NO2 | 115.06 | 1.75 | [M+H]+1 | Amino acid | Placenta hominis |
| 14 | Oxypeucedan hydrate | C16H16O6 | 304.09 | 30.61 | [M+H]+1 | Coumarins | Angelica dahurica |
| 15 | Ononin | C22H22O9 | 430.13 | 29.04 | [M+H]+1 | Flavonoids | Astragalus membranaceus |
| 16 | Morin | C15H10O7 | 302.04 | 26.64 | [M+H]+1 | Flavonoids | Carthamus tinctorius |
| 17 | Daidzein | C15H10O4 | 254.06 | 30.05 | [M+H]+1 | Flavonoids | Astragalus membranaceus |
| 18 | Calycosin | C16H12O5 | 284.07 | 30.81 | [M+H]+1 | Flavonoids | Astragalus membranaceus |
| 19 | Epicatechin | C15H14O6 | 290.08 | 24.09 | [M-H]-1 | Flavonoids | Astragalus membranaceus |
| 20 | Quercetin | C15H10O7 | 302.04 | 30.93 | [M-H]-1 | Flavonoids | Glehnia littoralis |
| 21 | Taurine | C2H7NO3S | 125.01 | 1.60 | [M-H]-1 | Ketones | Hirudo nipponia |
| 22 | Maleic acid | C4H4O4 | 116.01 | 1.80 | [M-H]-1 | Ketones | Prunus persica |
| 23 | Nicotinic acid | C_6_H_5_NO_2_ | 123.03 | 3.05 | [M+H]+1 | Ketones | Astragalus membranaceus |
| 24 | Azelaic acid | C9H16O4 | 188.10 | 28.23 | [M-H]-1 | Ketones | Prunus persica |
| 25 | Gallic acid | C7H6O5 | 170.02 | 8.54 | [M-H]-1 | Phenols | Paeonia lactiflora |
| 26 | Protocatechualdehyde | C7H6O3 | 138.03 | 23.35 | [M-H]-1 | Phenols | Carthamus tinctorius |
| 27 | p-Hydroxybenzaldehyde | C7H6O2 | 122.04 | 25.02 | [M-H]-1 | Phenols | Paeonia lactiflora |
| 28 | Medicarpin | C16H14O4 | 270.09 | 38.59 | [M-H]-1 | Phenols | Astragalus membranaceus |
| 29 | Marmesin | C14H14O4 | 246.09 | 26.79 | [M-H]-1 | Phenylpropanoids | Glehnia littoralis |
| 30 | Cinnamaldehyde | C9H8O | 132.06 | 28.03 | [M+H]+1 | Phenylpropanoids | Curcuma wenyujin |
| 31 | Caffeic acid | C9H8O4 | 180.04 | 24.75 | [M-H]-1 | Phenylpropanoids | Astragalus membranaceus |
| 32 | Suberosin | C15H16O3 | 244.11 | 42.79 | [M-H]-1 | Phenylpropanoids | Angelica dahurica |
| 33 | p-Coumaric acid | C9H8O3 | 164.05 | 26.34 | [M-H]-1 | Phenylpropanoids | Curcuma wenyujin |
| 34 | Atractylenolide I | C15H20O2 | 232.17 | 28.75 | [M+H]+1 | Terpenoids | Glehnia littoralis |
| 35 | Albiflorin | C23H28O11 | 480.16 | 28.02 | [M+FA-H]-1 | Terpenoids | Paeonia lactiflora |
| 36 | Curcumenol | C15H22O2 | 234.16 | 34.05 | [M+H]+1 | Terpenoids | Curcuma wenyujin |
| 37 | Parthenolide | C15H20O3 | 248.14 | 35.77 | [M+H-H2O]+1 | Terpenoids | Carthamus tinctorius |
| 38 | Paeoniflorin | C23H28O12 | 469.16 | 23.83 | [M-H]-1 | Terpenoids | Paeonia lactiflora |
| 39 | Atractylenolide II | C15H20O2 | 232.15 | 28.75 | [M+H]+1 | Terpenoids | Carthamus tinctorius |
