# Supplementary material for: Huayu Wan induces ferroptosis and enhances anti-PD-1 efficacy in cisplatin-resistant esophageal squamous cell carcinoma
Source: Front Immunol. 2026 Oct 27;17:1967436. doi: 10.3389/fimmu.2026.1967436 (PMC13747869; doi:10.3389/fimmu.2026.1967436)
Supplement: Supplementary file 4 [file Table4.docx]

**Table s4**. Potential targetgenes of bioactive compounds in HYW for ESCC.

| NO. | symbol | entrezID | NO. | symbol | entrezID | NO. | symbol | entrezID | NO. | symbol | entrezID |
| --- | --- | --- | --- | --- | --- | --- | --- | --- | --- | --- | --- |
| 1 | PTGS1 | 5742 | 50 | MT2A | 4502 | 99 | CD14 | 929 | 148 | PARP1 | 142 |
| 2 | KCNH2 | 3757 | 51 | MKI67 | 4288 | 100 | TYR | 7299 | 149 | COL3A1 | 1281 |
| 3 | PTGS2 | 5743 | 52 | EP300 | 2033 | 101 | ODC1 | 4953 | 150 | CXCL11 | 6373 |
| 4 | KDR | 3791 | 53 | TFF1 | 7031 | 102 | MMP3 | 4314 | 151 | CXCL2 | 2920 |
| 5 | ADRB2 | 154 | 54 | TRPM2 | 7226 | 103 | CCND1 | 595 | 152 | CHEK2 | 11200 |
| 6 | HSP90AA1 | 3320 | 55 | GH1 | 2688 | 104 | BCL2 | 596 | 153 | CLDN4 | 1364 |
| 7 | NCOA1 | 8648 | 56 | GHR | 2690 | 105 | BCL2L1 | 598 | 154 | PPARD | 5467 |
| 8 | SLC6A3 | 6531 | 57 | TGFB1I1 | 7041 | 106 | MMP2 | 4313 | 155 | HSF1 | 3297 |
| 9 | EGFR | 1956 | 58 | P4HB | 5034 | 107 | MMP9 | 4318 | 156 | CXCL10 | 3627 |
| 10 | VEGFA | 7422 | 59 | CYP21A2 | 1589 | 108 | MAPK1 | 5594 | 157 | CHUK | 1147 |
| 11 | TNF | 7124 | 60 | GADD45A | 1647 | 109 | IL10RA | 3587 | 158 | SPP1 | 6696 |
| 12 | IL6R | 3570 | 61 | BARD1 | 580 | 110 | EGF | 1950 | 159 | RUNX2 | 860 |
| 13 | IL1B | 3553 | 62 | BAP1 | 8314 | 111 | RB1 | 5925 | 160 | RASSF1 | 11186 |
| 14 | PGF | 5228 | 63 | RAD51 | 5888 | 112 | ELK1 | 2002 | 161 | E2F1 | 1869 |
| 15 | ESR1 | 2099 | 64 | AKT1 | 207 | 113 | NFKBIA | 4792 | 162 | E2F2 | 1870 |
| 16 | AR | 367 | 65 | PLAU | 5328 | 114 | CASP8 | 841 | 163 | ACP3 | 55 |
| 17 | PPARG | 5468 | 66 | POR | 5447 | 115 | RAF1 | 5894 | 164 | IGFBP3 | 3486 |
| 18 | RXRA | 6256 | 67 | COMT | 1312 | 116 | SOD1 | 6647 | 165 | IGF2 | 3481 |
| 19 | ESR2 | 2100 | 68 | CREB1 | 1385 | 117 | PRKCA | 5578 | 166 | CD40LG | 959 |
| 20 | DPP4 | 1803 | 69 | CCL2 | 6347 | 118 | MMP1 | 4312 | 167 | IRF1 | 3659 |
| 21 | MAPK14 | 1432 | 70 | IL2RA | 3559 | 119 | HIF1A | 3091 | 168 | ERBB3 | 2065 |
| 22 | GSK3B | 2932 | 71 | PLAT | 5327 | 120 | RUNX1T1 | 862 | 169 | PON1 | 5444 |
| 23 | CDK2 | 1017 | 72 | GRIN1 | 2902 | 121 | CDK1 | 983 | 170 | HK2 | 3099 |
| 24 | CHEK1 | 1111 | 73 | IL1A | 3552 | 122 | HSPA5 | 3309 | 171 | RASA1 | 5921 |
| 25 | CCNA2 | 890 | 74 | GCLC | 2729 | 123 | ERBB2 | 2064 | 172 | GSTM1 | 2944 |
| 26 | NCOA2 | 10499 | 75 | GSS | 2937 | 124 | ACACA | 31 |  |  |  |
| 27 | CA2 | 760 | 76 | CYP19A1 | 1588 | 125 | HMOX1 | 3162 |  |  |  |
| 28 | DRD2 | 1813 | 77 | ABCG2 | 9429 | 126 | CYP1A2 | 1544 |  |  |  |
| 29 | RXRB | 6257 | 78 | ACE | 1636 | 127 | MYC | 4609 |  |  |  |
| 30 | RELA | 5970 | 79 | HAS2 | 3037 | 128 | F3 | 2152 |  |  |  |
| 31 | FOS | 2353 | 80 | PGR | 5241 | 129 | GJA1 | 2697 |  |  |  |
| 32 | CDKN1A | 1026 | 81 | CASP9 | 842 | 130 | CYP1A1 | 1543 |  |  |  |
| 33 | EIF6 | 3692 | 82 | FASN | 2194 | 131 | SELE | 6401 |  |  |  |
| 34 | BAX | 581 | 83 | FASLG | 356 | 132 | CXCL8 | 3576 |  |  |  |
| 35 | CASP3 | 836 | 84 | CTSD | 1509 | 133 | PRKCB | 5579 |  |  |  |
| 36 | TP53 | 7157 | 85 | ADH1B | 125 | 134 | BIRC5 | 332 |  |  |  |
| 37 | LDLR | 3949 | 86 | ADH1C | 126 | 135 | HSPB1 | 3315 |  |  |  |
| 38 | CAT | 847 | 87 | TPI1 | 7167 | 136 | NR1I2 | 8856 |  |  |  |
| 39 | IGF1R | 3480 | 88 | MAPK10 | 5602 | 137 | CYP1B1 | 1545 |  |  |  |
| 40 | STAT1 | 6772 | 89 | TOP1 | 7150 | 138 | CCNB1 | 891 |  |  |  |
| 41 | CYP3A4 | 1576 | 90 | EDNRA | 1909 | 139 | THBD | 7056 |  |  |  |
| 42 | CAV1 | 857 | 91 | ABCB1 | 5243 | 140 | SERPINE1 | 5054 |  |  |  |
| 43 | ICAM1 | 3383 | 92 | ALOX5 | 240 | 141 | COL1A1 | 1277 |  |  |  |
| 44 | APOB | 338 | 93 | CD36 | 948 | 142 | IFNG | 3458 |  |  |  |
| 45 | VCAM1 | 7412 | 94 | LPL | 4023 | 143 | MPO | 4353 |  |  |  |
| 46 | ECE1 | 1889 | 95 | PPARA | 5465 | 144 | TOP2A | 7153 |  |  |  |
| 47 | IL4R | 3566 | 96 | HP | 3240 | 145 | GSTP1 | 2950 |  |  |  |
| 48 | AHR | 196 | 97 | PPARGC1A | 10891 | 146 | NFE2L2 | 4780 |  |  |  |
| 49 | RHOA | 387 | 98 | ACHE | 43 | 147 | NQO1 | 1728 |  |  |  |
